# Integrative single-cell and machine learning analysis predicts lactylation-driven therapy resistance in prostate cancer: a molecular docking and experiments-validated framework for treatment optimization

**DOI:** 10.3389/fimmu.2025.1647384

**Published:** 2025-09-02

**Authors:** Zhiyu Liu, Yuqi Li, Juan Wang, Yang Zeng, Qilong Wu, Xinyao Zhu, Tao Zhou, Qingfu Deng

**Affiliations:** ^1^ Department of Urology, Affiliated Hospital of Southwest Medical University, Luzhou, Sichuan, China; ^2^ Public Center of Experimental Technology, Southwest Medical University, Luzhou, Sichuan, China; ^3^ Department of Urology, Santai Hospital Affiliated to North Sichuan Medical College, Mianyang, Sichuan, China

**Keywords:** prostate adenocarcinoma, drug resistance, lactylation, single-cell sequencing, machine learning, biomarkers

## Abstract

**Background:**

Prostate cancer (PCa) is a common malignancy in males. Predicting its prognosis and addressing drug resistance remain challenging. This study develops a novel prognostic model focusing on lactylation and resistance, which plays a crucial role in tumor biology.

**Methods:**

Single-cell analysis was employed to identify subpopulations expressing lactylation-related genes. Transcriptomic sequencing was used to identify drug resistance-associated genes. Univariate Cox proportional hazards models and machine learning techniques were used to identify prognostic genes, assisting in the development of a risk assessment framework. Additionally, we investigated how features related to lactylation and drug resistance correlate with clinical characteristics, the tumor microenvironment, and treatment responses, revealing potential interconnections.

**Results:**

In this study, a model composed of 29 biomarkers was developed by integrating single-cell data and machine learning algorithms. The model predictive efficacy was validated through Kaplan-Meier (KM) analysis, univariate Cox (HR=3.59, 95%CI: 2.78-4.63) and multivariate Cox (HR=2.81, 95%CI: 1.96-4.03) regression. Comprehensive analysis revealed significant differences in tumor immune dysfunction and exclusion (TIDE) scores, immunophenoscore (IPS) scores, and chemotherapy drug sensitivity between high-risk and low-risk groups, suggesting that specific biomarkers may be closely associated with prognosis. Furthermore, molecular docking analysis and experiments were conducted to explore the relationship between drug resistance and risk gene-encoded proteins.

**Conclusions:**

The prognostic model effectively predicts the progression-free interval (PFI) and drug response, with accurate risk stratification for PCa patients. Our findings highlight the potential of risk genes in the development of personalized treatment strategies and enhancing PCa prognostic assessment.

## Introduction

Prostate cancer (PCa) is one of the most prevalent malignant tumors globally, characterized by steadily climbing diagnosis and fatality rates that have established it as a critical public health concern for male health (1). Despite substantial progress in early diagnosis and therapeutic interventions, the heterogeneity and complexity of PCa remain formidable challenges in prognosis prediction and treatment decision-making. Targeted therapeutic agents, including abiraterone acetate, bicalutamide, and enzalutamide, have markedly improved patient outcomes. However, individual responses exhibit significant variability, and some patients will progress to castration-resistant prostate cancer (CRPC), developing resistance to novel endocrine therapies (2–4). Conventional clinicopathological indicators, such as the Gleason score and prostate-specific antigen (PSA) levels, provide limited prognostic accuracy and fail to comprehensively assess disease risk and therapeutic response, making them insufficient for personalized treatment strategies (5, 6). Consequently, the development of prognostic models for CRPC based on molecular characteristics is essential for enhancing survival rates and optimizing treatment outcomes in PCa patients.

Lactylation is a recently identified post-translational protein modification that functions as a key metabolic regulator in cancer biology. It involves the covalent attachment of lactyl groups to histones or other proteins, thereby influencing gene expression and cellular functions. As a result, lactylation is implicated in key biological processes of cancer cells, including proliferation, invasion, treatment resistance, and immune evasion (7–10). Consequently, exploring the functions of lactylation-related genes in PCa could provide novel insights into tumor metabolic regulation and facilitate the identification of molecular biomarkers for prognostic model development.

Lactylation has emerged as a critical metabolic regulatory mechanism in cancer biology and is closely linked to the progression of PCa. This lactate-driven post-translational modification modulates gene expression by covalently modifying histones like H3K18la—as well as key signaling proteins (11). In PCa, elevated glycolytic activity results in excessive lactate production, a hallmark of tumor aggressiveness. This accumulation of lactate may facilitate epigenetic reprogramming by altering the chromatin accessibility of neuronal genes, thereby contributing to therapeutic resistance and neuroendocrine differentiation (12, 13). Increasing evidence indicates that lactylation levels are positively associated with resistance to multiple chemotherapeutic agents, underscoring its potential as both a prognostic biomarker and a therapeutic target (10, 14, 15). In the present study, we systematically assessed the prognostic significance of lactylation using single-cell transcriptomic analysis combined with machine learning techniques, thereby bridging mechanistic insights with potential clinical applications.

With the exponential progression of high-throughput sequencing technologies, transcriptomic sequencing data has provided crucial insights into the mechanisms underlying PCa (16). Furthermore, the emergence of single-cell sequencing technology has revolutionized tumor microenvironment characterization, offering novel perspectives on tumor progression and mechanisms of drug resistance (17, 18).

This study integrates transcriptomic sequencing and single-cell data to develop a prognostic model for PCa, with the goal of improving the accuracy of patient risk stratification. Furthermore, drug sensitivity analysis, tumor immune dysfunction and exclusion (TIDE) scores, and immunophenoscore (IPS) are employed to assess differences in responses to targeted therapy and immunotherapy among patients in distinct risk groups. By identifying novel biomarkers and establishing a theoretical framework for personalized PCa treatment, this research contributes to the advancement of precision medicine.

## Materials and methods

### Acquisition of transcriptomic data

The public genomic and transcriptomic profiles of PCa, along with the associated clinical data, were sourced from UCSC Xena (https://xenabrowser.net) and the study by Jianfang Liu, Tara Lichtenberg, et al. (19). The single nucleotide variant (SNV) data were retrieved from The Cancer Genome Atlas (TCGA) (https://portal.gdc.cancer.gov). The analysis comprised 495 prostate adenocarcinoma (PRAD) samples, all of which had available survival data and were used for survival-related analysis. The validation dataset and prognostic data were primarily obtained from the PCaDB database (http://bioinfo.jialab-ucr.org/PCaDB).

### Collection of single-cell sequencing

The single-cell dataset GSE206962 was downloaded from the Gene Expression Omnibus (GEO) database (https://www.ncbi.nlm.nih.gov/geo) and analyzed using the R package “Seurat” (version 5.1.0) (20). A cohort analysis was conducted on four patient samples. Gene expression counts for selected cells ranged from 500 to 5000, with a mitochondrial gene proportion of less than 10% (**Supplementary Figure 2**). The top 2000 variable genes were normalized and selected using the “NormalizeData” and “FindVariableFeatures” functions in Seurat. After preprocessing the single-cell gene expression profiles, principal component analysis (PCA) was performed for dimensionality reduction, followed by batch effect correction using the “RunHarmony” function from the “harmony” R package. Cell clustering was performed using the Louvain algorithm on the k-nearest neighbor (KNN) graph. t-SNE analysis was performed with a resolution of 0.8, generating 15 principal components and 24 clusters. Cell clusters were classified based on cell-type-specific markers (B cells: CD79A, IGHG1; T cells: CD3D, CD3E, CD2; macrophages: CD68, C1QA, C1QB; fibroblasts: DCN, COL3A1, RGS5; endothelial cells: VWF, PECAM1, ENG; epithelial cells: EPCAM, KRT8, KRT18), and a quantitative analysis was performed on the proportions of different cell types.

### Single-cell data scoring

To assess the expression characteristics and functional significance of lactylation-related gene sets at the single-cell level in prostate cancer, we performed a comprehensive analysis of single-cell data using multiple gene set scoring algorithms. Specifically, we employed four algorithms—AUCell, UCell, AddModuleScore, and ssGSEA—to evaluate the activity of lactylation-related gene sets in individual cells (21–24). The selection of these algorithms is based on their unique advantages in addressing the challenges associated with single-cell analysis. AUCell detects subtle gene set enrichment based on gene expression levels. UCell performs cell-wise scoring independently, unaffected by random seeds or the number of cells included. AddModuleScore calculates gene module scores by subtracting the average expression of a background gene set from that of a target gene set. ssGSEA, on the other hand, considers gene–gene interactions and functional associations, thereby more accurately reflecting the activity of biological processes and pathways. By integrating these methods, we generated a score that enables cross-validation of lactylation signaling activity, thereby reducing the bias inherent in any single algorithm. The scores from each algorithm were then averaged for each cell to generate a composite score for the lactylation-related gene set. Using the median composite score across all single cells as a threshold, we categorized the cells into active and non-active groups. The “FindMarkers” algorithm was then applied to identify marker genes in cells with high gene expression activity, based on the median score. The R package “ggpubr” was used to visualize the single-cell scores of each cell in tSNE plots, thereby aiding the identification of clusters with active gene expression.

### Development of a prognostic model

Data from cellular transcriptome sequencing, scRNA-seq, and TCGA were utilized to perform univariate Cox regression analysis for identifying prognostic-related genes (*p < 0.05*). Based on these genes, TCGA served as the training data, and external datasets were used for validation. To mitigate overfitting of MPT-driven necrosis-related prognostic genes, Least Absolute Shrinkage and Selection Operator (LASSO)-Cox regression was conducted using the R package “glmnet” to select significant genes and their corresponding regression coefficients (25). Hyperparameter tuning was performed via 10-fold cross-validation with the penalty parameter (λ) selected from 100 candidate values logarithmically spaced. The optimal λ was determined by minimizing the partial likelihood deviance under the “1-standard-error rule” (1SE), ensuring a parsimonious model with robust generalizability. The risk score for PRAD patients was calculated using the formula: Risk Score=Σ (gene expression × regression coefficient) , where Σ represents the sum of all selected genes, gene expression denotes the expression level, and the regression coefficient reflects the weight assigned to each gene. Patients were classified into low-risk and high-risk groups based on the median risk score. Several statistical methods were employed to assess the features, including the log-rank test from the R package “survival”, Kaplan-Meier (KM) analysis to evaluate the progression-free interval (PFI) differences between two groups, and histograms to assess risk score differences across clinical subgroups.

### Construction of a nomogram

To identify whether the risk score can act as an independent prognostic factor, we conducted both univariate and multivariate Cox regression analyses. Furthermore, utilizing the TCGA-PRAD dataset, we applied the R package “rms” to develop a nomogram that integrates risk scores with clinical and pathological features (26). The nomogram was designed to predict PFI outcomes at different years.

### Enrichment analysis

Differential gene expression analysis was conducted using count data from the TCGA-PRAD cohort, employing the R package DESeq2. Stringent filtering thresholds were applied, including an adjusted p-value of less than 0.05 and |log2FC|>1. As a result, a set of differentially expressed genes (DEGs) was identified between the high-risk and low-risk patient groups. Then functional enrichment analysis was conducted on differentially expressed genes in the cohort to annotate biological pathways and examine enrichment patterns. Gene Ontology (GO) and Kyoto Encyclopedia of Genes and Genomes (KEGG) enrichment analyses, as well as Gene Set Enrichment Analysis (GSEA), were performed using the R package “clusterProfiler” (27).

### Tumor mutational burden analysis

The R package “maftools” was utilized to organize and visualize somatic mutations in TCGA-PRAD, including single nucleotide polymorphisms (SNPs), insertions and deletions (INDELs), tumor mutational burden (TMB), and mutation frequencies (28).

### Evaluation of drug treatment

To evaluate the potential differences in responses to targeted therapies and immunotherapies between patients in two groups, we conducted a comprehensive analysis using multiple drug sensitivity tools and databases. Initially, we assessed the tumor immune dysfunction and exclusion characteristics of both groups using TIDE data, which predicted their responses to immune checkpoint inhibitors. Subsequently, we calculated the IPS using the The Cancer Immunome Atlas (TCIA) database (https://tcia.at/home) to further validate the differences in tumor responses to various targeted therapies, with a focus on anti-CTLA-4 and anti-PD-1 therapies (29). Furthermore, we integrated three drug sensitivity datasets, including Genomics of Drug Sensitivity in Cancer (GDSC) 1.0, GDSC2.0, and Cancer Therapeutics Response Portal (CTRP) to analyze sensitivity to various chemotherapeutic agents between two risk groups (30, 31).

### Molecular docking

Molecular docking analysis was to explore the potential binding interactions between enzalutamide and the identified target genes. The molecular structure of enzalutamide was retrieved from PubChem (https://pubchem.ncbi.nlm.nih.gov), while the protein structures of the risk genes were predicted using the AlphaFold server (https://alphafoldserver.com). Then we performed molecular docking using the CB-DOCK2 online platform (https://cadd.labshare.cn/cb-dock2/php/blinddock.php), and the model exhibiting the lowest binding energy was selected as the optimal result. A binding energy lower than -7 kJ/mol was considered indicative of a stable interaction (32). Lastly, a heatmap depicting the binding energies between the risk genes and enzalutamide was generated using an online platform (https://www.bioinformatics.com.cn) (33).

### Cell lines and cell culture

The LNCaP (CL-0143), and C4-2 (iCell-h626) cell lines were obtained from Procell Life Science & Technology Co., Ltd. (Wuhan, China) and Cellverse Co., Ltd. (Shanghai, China), while the normal prostate epithelial cell line RWPE-1 (BNCC341583) was sourced from BeNa Culture Collection (Langfang, China). All cell lines were authenticated by short tandem repeat (STR) profiling and confirmed to be free of mycoplasma contamination. LNCaP and C4–2 cells were cultured in RPMI 1640 medium. RWPE-1 cells were cultured in keratinocyte medium supplemented with 1% keratinocyte growth supplement (KGS) and 1% P/S.

To establish enzalutamide-resistant cell lines, we continuously cultured LNCaP cells and C4–2 cells in a medium supplemented with enzalutamide (GLPBIO, California, USA) for a duration of four months. During this period, the cells were subjected to incrementally increasing concentrations of enzalutamide (ranging from 5 to 15 µM). The resultant resistant cells were maintained in a medium containing 5 µM enzalutamide and were designated as LNCaP-Enza cells and C4-2-Enza cells. These cells were cultured in an atmosphere containing 5% CO2 at a temperature of 37 °C.

### Cell transfection

LNCaP-Enza and C4-2-Enza cell lines were transfected with 100nM of targeted small interfering RNA (siRNA) (siDEGS1-1: UAAUGCAACUGCCAAACGCTT, GCGUUUGGCAGUUGCAUUATT; siDEGS1-2: GAGCAUUACAUGUUCUUAATT, UUAAGAACAUGUAAUGCUCTT) were obtained from FENGHUISHENGWU in a medium without P/S. Twenty-four hours post-transfection, the cells were further cultured in standard complete medium supplemented with 10% FBS and 1% P/S.

### Half-maximal inhibitory concentration assay

LNCaP-Wt, C4-2-Wt, as well as transfected or non-transfected siRNA LNCaP-Enza and C4-2-Enza cells were seeded into 24-well plates. The cells were treated with different concentrations of enzalutamide for 48 hours. Then serum-free medium containing 10% CCK-8 solution was added to each well, followed by incubation for 2 hours at 37°C. Subsequently, the absorbance at 450 nm was measured. Each group had three replicate wells, and the experiment was performed in triplicate.

### RNA extraction and quantitative real-time polymerase chain reaction

Total RNA was extracted from LNCaP-Enza and C4-2-Enza cells (untreated or treated with Oxamate for 24 hours) using the Cell Total RNA Isolation Kit (FOREGENE, Chengdu, China). Total RNA was efficiently extracted from cultured cells using a spin-column-based method. After complete removal of culture medium, an appropriate volume of Buffer cRL1 was added for cell lysis. The lysate was then mixed with Buffer cRL2 at a 1:1.6 volume ratio and centrifuged through a DNA-Cleaning Column to remove genomic DNA. The supernatant was subsequently loaded onto an RNA-Only Column in batches, followed by sequential washing with Buffer RW1 (500 μL) and Buffer RW2 (700 μL, twice) to remove impurities. Finally, RNA was eluted using 60 μL of RNase-Free ddH_2_O preheated to 65°C. All procedures were performed at room temperature.

RT-qPCR analysis was performed with SYBR Green qPCR Master Mix (TOYOBO, Shanghai, China). GAPDH served as the internal reference gene. Several primers were used to perform RT-qPCR analysis. The sequences of the gene-specific primers used are as follows: for the AR, the forward primer is TACCAGCTCACCAAGCTCCT and the reverse primer is GCTTCACTGGGTGTGGAAA; for the KLK3, the forward primer is GTCCGTGACGTGGATTGGTG and the reverse primer is AGACTGCCCTGCCACGAGA; for the FOLH1, the forward primer is TCAAGGAATGCCAGAGGGC and the reverse primer is CTGAAAACTTTCCCATATCTGGC. The levels of mRNA were measured utilizing the 2−ΔΔCT method. Samples were analyzed in triplicates.

### Western blot analysis

Total protein extraction was carried out using RIPA Lysis Buffer, supplemented with 1× Protease Inhibitor Cocktail and 1× Phosphatase Inhibitor Cocktail (APEXBIO, Houston, USA). Protein concentrations were quantified using the Bicinchoninic Acid (BCA) Protein Assay Kit (CWBIO, Jiangsu, China). The proteins were separated by sodium dodecyl sulfate-polyacrylamide gel electrophoresis (SDS-PAGE) on a 7.5% gel (Epizyme, Shanghai, China). Subsequently, the separated proteins were transferred onto a polyvinylidene difluoride (PVDF) membrane (0.45 µm, Merck Millipore, Germany). Following the transfer, the membrane was blocked and incubated with primary antibodies. After washing, the bound antibodies were detected using Super ECL Plus Western Blotting Substrate (BIOGROUND, Chongqing, China) on a Western blot imaging system (Tanon 5200CE, Shanghai, China). β-Tublin served as an internal control for normalization. Western blot analyses were performed in triplicate.

### Colony-formation assay

Following transfection, the enzalutamide-resistant cell lines LNCaP-Enza and C4-2-Enza were plated in six-well plates at densities of 5000 cells per well, respectively, and were then cultured continuously for a period of 10 to 20 days. Following this incubation period, the cells were fixed and subsequently stained with a 0.1% crystal violet solution (Beyotime, Jiangsu, China).

### EdU detects the proliferation of cells

LNCaP-Enza and C4-2-Enza cells were seeded into 6-well plates. When the cell density reached 80%, EdU (Cy5) was diluted to a final concentration of 10 μM in RPMI 1640 medium supplemented with 10% fetal bovine serum (FBS). The cells were incubated for 5 hours. Following fixation removal, the cells were washed twice with 3% BSA solution for 5 minutes each. Subsequently, the cells were permeabilized with 0.3% Triton solution and incubated at room temperature for 15 minutes. A Click reaction solution was prepared by mixing 860 μL of 1× EdU Reaction Buffer, 40 μL of CuSO4, 1 μL of Cy5 azide, and 100 μL of 1× EdU Buffer Additive per milliliter. The Click reaction solution was added to each well and incubated in the dark at room temperature for 30 minutes. After incubation, Hoechst 33342 was added to a final concentration of 5 μL/mg, and the cells were incubated in the dark at room temperature for an additional 15 minutes. Finally, images of Cy5 azide and Hoechst 33342 were respectively captured using a confocal microscope with excitation wavelengths of 646 nm and 350 nm.

### Glucose and lactate assays

LNCaP-Enza and C4-2-Enza cells were seeded into 6-well plates. Once the cells reached approximately 50% confluency, they were transfected with siRNA. Glucose and lactate concentrations were subsequently quantified using the Glucose Content Assay Kit (Sangon Biotech, D799408) and the Lactic Acid (L-LA) Content Assay Kit (Sangon Biotech, D799099), respectively, according to the manufacturers’ protocols.

### Statistical analysis

Statistical analyses were performed using R (version 4.4.1) and RStudio (version 2023.12.1 + 402), along with GraphPad Prism software (version 10.1.2). The KM method was applied for survival analysis. Univariate and multivariate Cox regression analyses were conducted to assess the prognostic significance and independence of the risk score. Receiver operating characteristic (ROC) curves were utilized to evaluate the robustness of the PFI prognostic model. The Wilcoxon test was employed to compare differences in risk scores, drug treatment scores, and drug sensitivity between different groups within the TCGA-PRAD cohort. Experimental data are presented as mean ± standard deviation. Comparisons of means between two groups were conducted using the t-test, while comparisons among multiple groups utilized one-way analysis of variance (ANOVA). A p.value of < 0.05 was considered statistically significant, unless otherwise specified; *p < 0.05, **p < 0.01, ***p < 0.001, ****p < 0.0001.

## Results

### Single-cell sequencing analysis and gene set scoring

**Figure 1:**
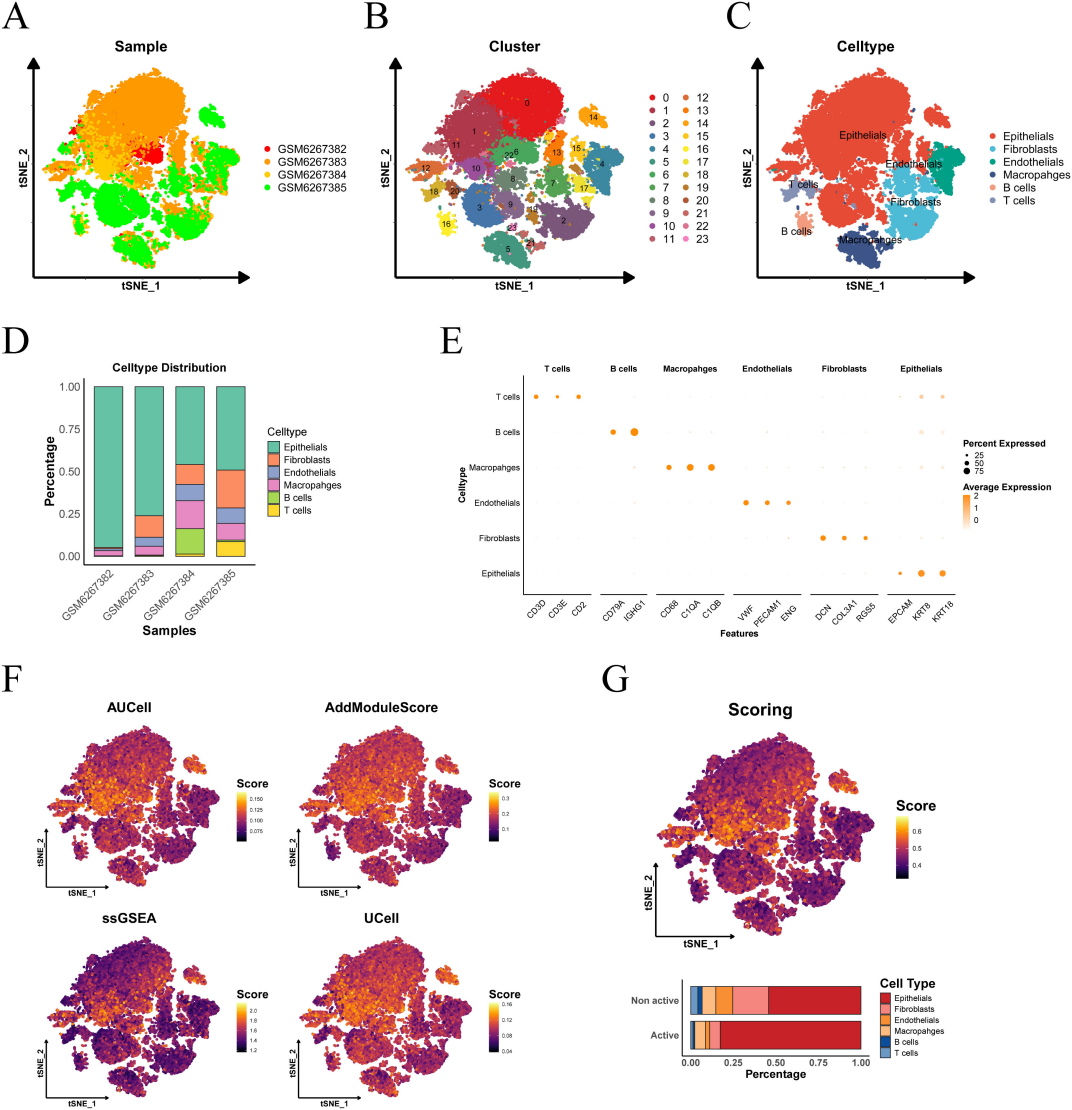
Cell type determination and scoring in single-cell sequencing. **(A)** tSNE visualization of PCa sample distribution. **(B)** Cellular cluster mapping within tSNE space. **(C)** Subpopulation annotation across tSNE coordinates. **(D)** Cellular composition profiling. **(E)** Cell-type-specific marker gene signatures. **(F)** tSNE heatmap depicting the scores from four single-cell scoring algorithms, with higher brightness corresponding to higher scores. **(G)** Proportion of cell subgroups in the active and inactive groups, as shown in the histogram.

The workflow of this study is depicted in **Supplementary Figure 1**. The PCa single-cell dataset (GSE206962) was obtained from the GEO database, which includes four cancer samples. The distribution of cells across samples was relatively uniform, suggesting no significant batch effects between samples (**Figure 1A**). Based on the gene expression profiles of the cells, 24 distinct clusters were identified (**Figure 1B**). Cell types were annotated using cell-specific markers, revealing six distinct cell types: T cells, B cells, macrophages, fibroblasts, endothelial cells, and epithelial cells (**Figure 1C**). A bar chart illustrates the proportion of each cell type within each PCa sample (**Figure 1D**). A dot plot highlights the marker genes associated with each cell type (**Figure 1E**).

Subsequently, we applied four single-cell scoring algorithms—AUCell, UCell, AddModuleScore, and ssGSEA—to assess the activity of lactylation-related genes. Among the four algorithms, epithelial cells consistently exhibited the highest scores (**Figures 1F, G**). The median of the average scores across all four methods was used as the threshold to classify the cells into active and inactive groups. In the lactylation-active group, epithelial cells accounted for the highest proportion, followed by macrophages and fibroblasts, indicating that the lactylation process predominantly occurs in prostate epithelial cells.

### Building a prognostic model through machine learning

**Figure 2:**
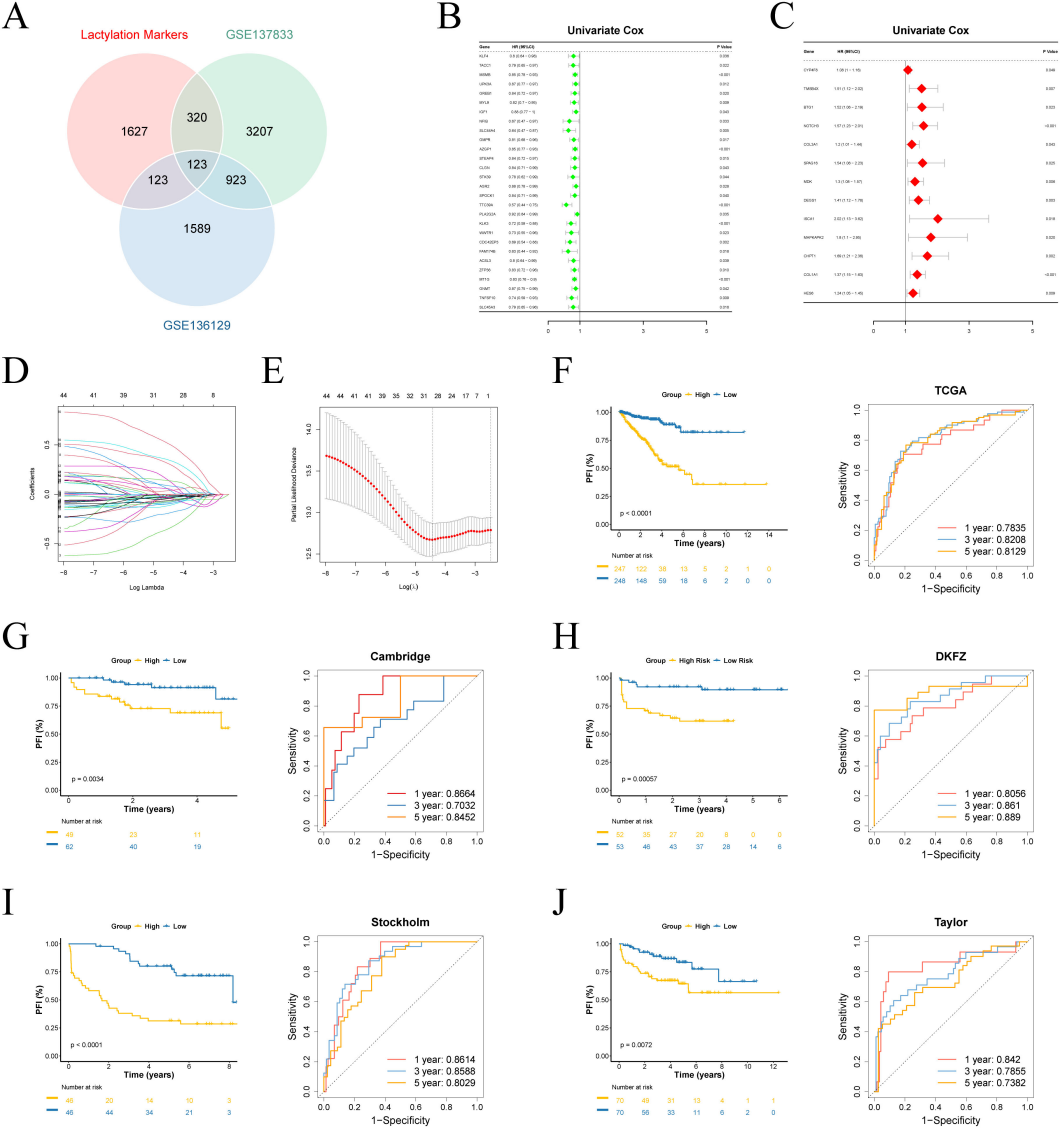
Construction of the prognostic model. **(A)** Venn analysis identifies overlapping genes among lactylation-active signatures, GSE137833 and GSE136129 differential expression profiles. **(B, C)** Univariate Cox analysis of survival-associated genes. **(D)** Visualization of Lasso coefficient trajectories. **(E)** The partial likelihood deviance with changing of log(λ). **(F-J)** Survival curves and time-dependent ROC curves for training and validation sets.

We performed differential analysis based on single-cell scoring to identify marker genes for the active group. These genes were then intersected with GSE137833 differential genes and GSE136129 differential genes, yielding a total of 123 genes (**Figure 2A**). Subsequently, univariate Cox regression analysis was conducted on these genes, which led to the identification of 45 prognostic-related genes (**Figures 2B, C**). Using these prognostic genes, we developed a predictive model with TCGA-PRAD as a training data, using LASSO regression analysis on the TCGA set was conducted to eliminate redundant genes, identifying 29 genes significantly associated with prognosis in patients and their coefficients (**Figures 2D, E**).

Utilizing a 29-gene expression signature, we established risk stratification for patients, dividing them into high- and low-risk categories using median risk scores as the threshold. Survival analysis demonstrated significant prognostic discrimination between risk groups in both TCGA and external validation cohorts (*p < 0.05*). Time-dependent ROC evaluation further validated the model’s robust predictive performance across four external datasets (**Figures 2F-J**). Furthermore, we evaluated the prognostic performance of the model in various other cancer types, where it demonstrated promising results in BLCA, BRCA, COAD, ESCA, KIRP, LUAD, and MESO (**Supplementary Figures 3A-J**).

**Figure 3:**
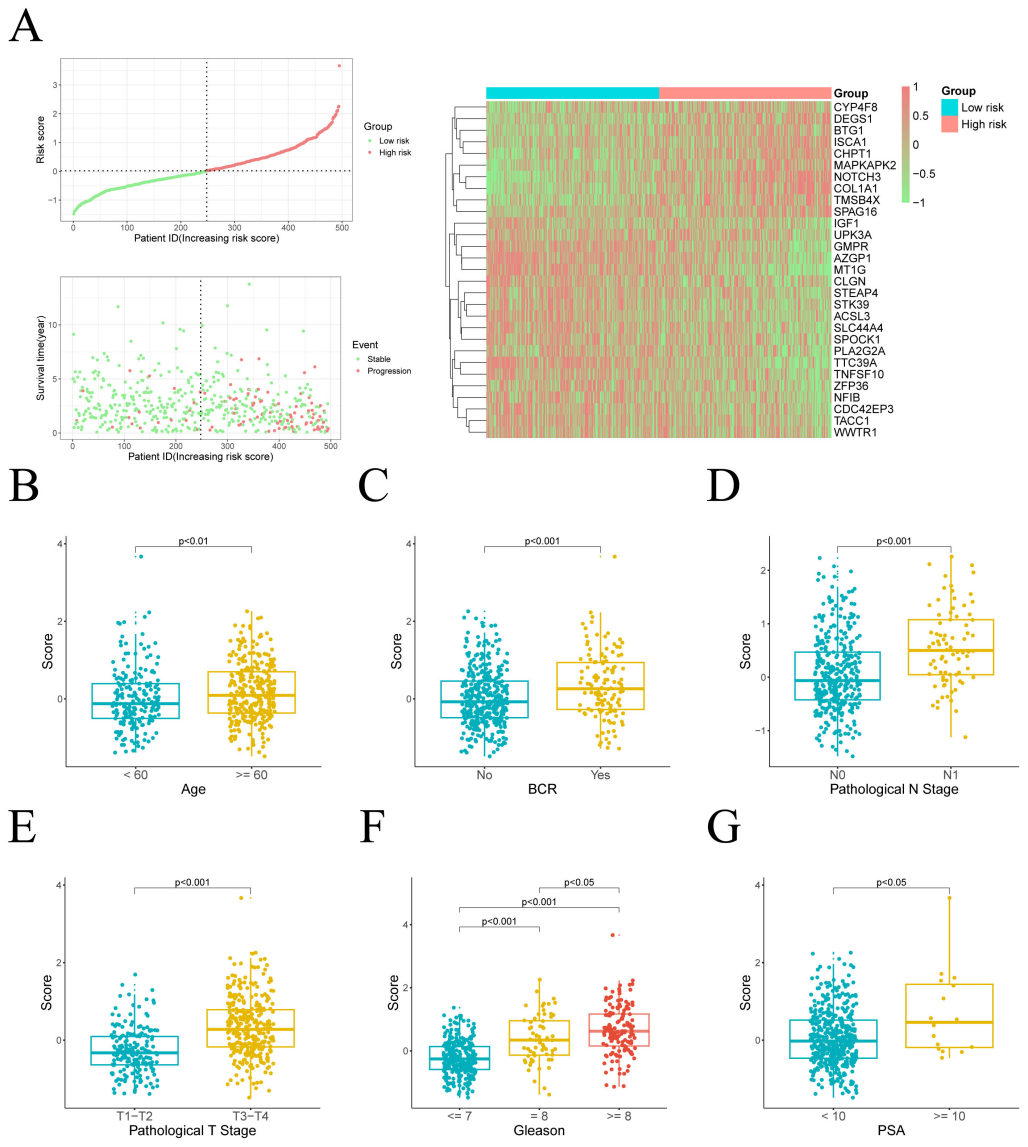
Distribution of risk scores and their association with clinical characteristics. **(A)** Left: The distribution of risk scores and PFI status for each patient. Right: A heatmap illustrating the expression of risk genes. **(B–G)** Comparison of risk scores across different clinical variable groups.

We further ranked the TCGA-PRAD samples according to their risk scores and generated scatter plots to visualize the survival status. This analysis demonstrated a positive correlation between the risk score and mortality rate in the patients from the TCGA cohort. The expression patterns of 29 prognostic genes were visualized through a heatmap, revealing distinct transcriptional profiles between risk-stratified groups (**Figure 3A**). Ten upregulated genes in high-risk patients potentially function as oncogenic drivers, while 19 elevated genes in low-risk patients may confer tumor-suppressive effects.

### Clinical feature prognostic analysis

We subsequently assessed the differences in risk scores across various clinical feature groups. Significant disparities in risk scores were observed between different ages, pathological T stages, pathological N stages, biochemical recurrence (BCR), PSA levels, and Gleason scores. Moreover, risk scores were positively correlated with the progression and severity of these clinical variables (**Figures 3B-G**).

**Figure 4:**
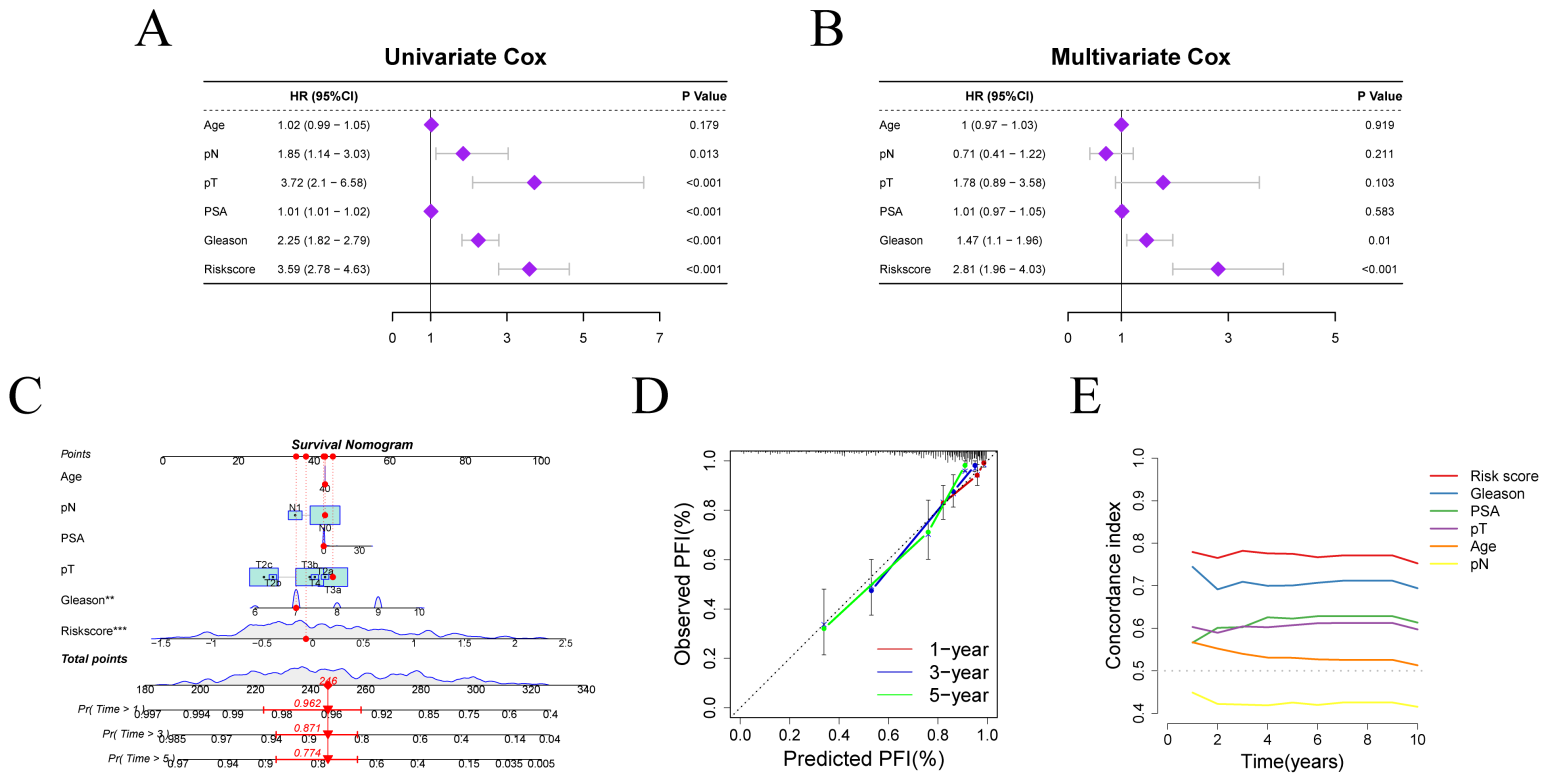
Independent prognostic factor analysis and nomogram construction. **(A, B)** Results of Cox regression analyses for risk scores and clinical characters. **(C)** Nomogram for predicting PFI in PRAD patients. **(D)** Calibration curve evaluating the accuracy of PFI prediction. **(E)** C-index evaluation of the risk score and clinical characters.

Given the notable differences in PFI and risk scores between two risk groups, we further explored the clinical prognostic value of the model. We assessed the risk score’s prognostic independence through correlation analysis with clinical features. Univariate and multivariate Cox regression analyses yielded hazard ratios (HRs) of 3.59 (95% CI: 2.78–4.63) and 2.81 (95% CI: 1.96–4.05), respectively, suggesting that the risk score may serve as an independent prognostic factor (**Figures 4A, B**). These findings suggest that the risk score is superior to other clinical characters in predicting patient prognosis.

We constructed a clinical nomogram incorporating age, pN, pT, PSA, Gleason score, and risk score for PFI prediction at 1-, 3-, and 5-year intervals (**Figure 4C**). The risk score emerged as the most influential prognostic indicator, highlighting the model’s predictive capacity for outcomes. Calibration analysis confirmed excellent agreement between predicted and observed PFI probabilities across different time points, validating the nomogram’s reliability (**Figure 4D**). Moreover, the concordance index (c-index) for the risk score outperformed that of other clinical variables, further confirming the superiority of our model in predicting patient survival prognosis relative to other clinical parameters (**Figure 4E**).

### Enrichment analysis

**Figure 5:**
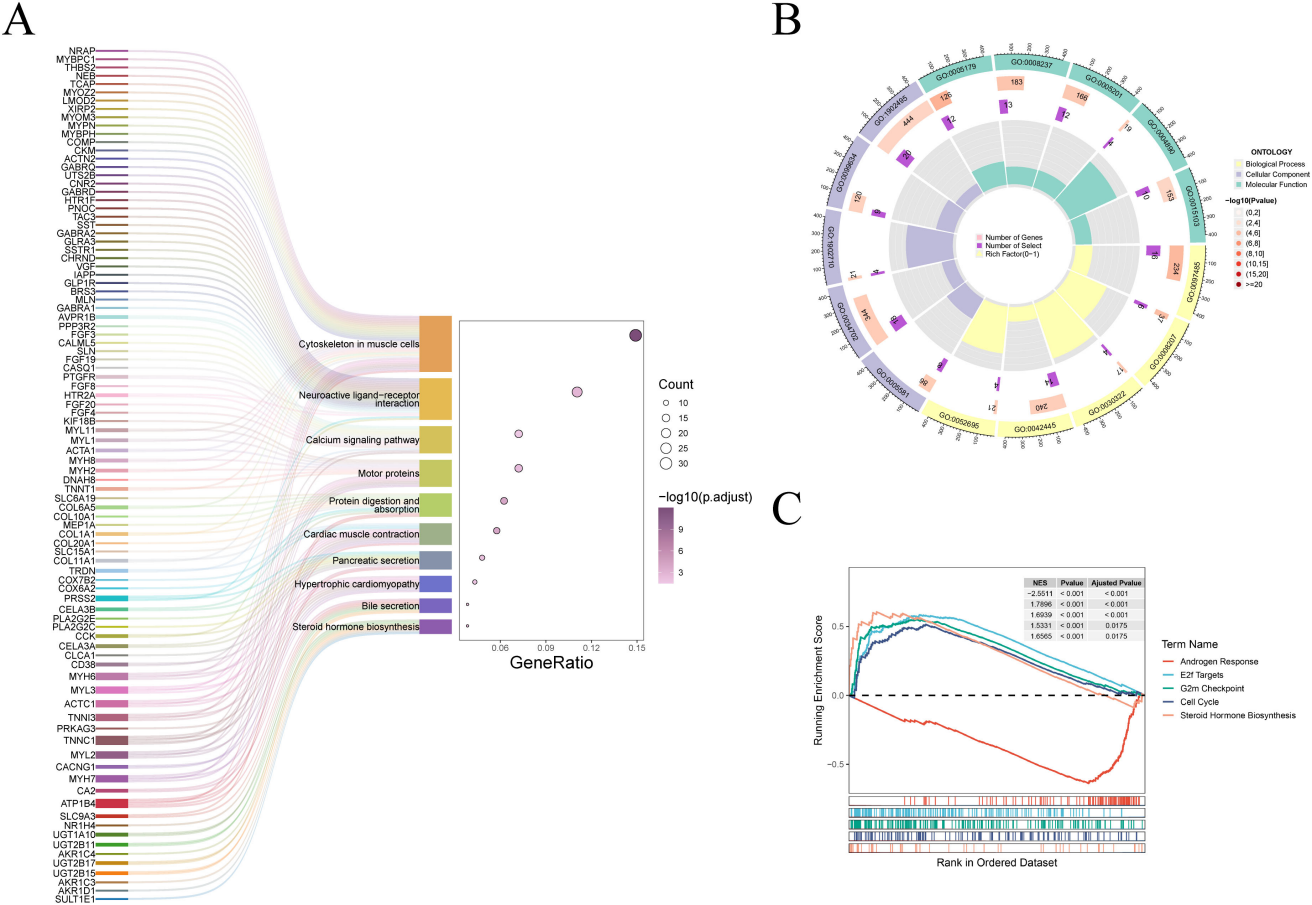
Enrichment analysis of different risk groups. **(A)** The Sankey diagram displays the KEGG pathway enrichment results. **(B)** The circular plot presents the results of the GO analysis. **(C)** The GSEA functional enrichment results for the high-risk and low-risk groups are shown.

To investigate the relationship between biological processes, signaling pathways, and risk scores, we conducted GO functional analysis and KEGG pathway enrichment analysis on differentially expressed genes (DEGs) between the high-risk and low-risk groups. DEGs were identified using a threshold of |log2FC| > 1 and *adjust.p.value < 0.05*. KEGG enrichment analysis revealed the significant involvement of the “Neuroactive ligand-receptor interaction”, “Steroid hormone biosynthesis” and “Calcium signaling pathway” (**Figure 5A**). GO analysis indicated that the significantly affected biological processes (BP) included “neuron projection guidance”, “C21-steroid hormone metabolic process”, “hormone metabolic process” and “cellular glucuronidation”. The most relevant cellular components (CC) involved were “monoatomic ion channel complex”, “GABA receptor complex” and “postsynaptic specialization membrane”, while the prominent molecular functions (MF) included “hormone activity”, “metallopeptidase activity” and “GABA-A receptor activity” (**Figure 5B**). GSEA revealed significant pathway differences between the low-risk and high-risk groups. The high-risk group exhibited increased activity in the “E2F targets,” “G2M checkpoint,” and “Cell cycle” pathways, whereas the low-risk group was predominantly enriched in the “Androgen response” and “Steroid hormone biosynthesis” pathways (**Figure 5C**).

### Tumor mutation burden and response to drug therapy evaluation

**Figure 6:**
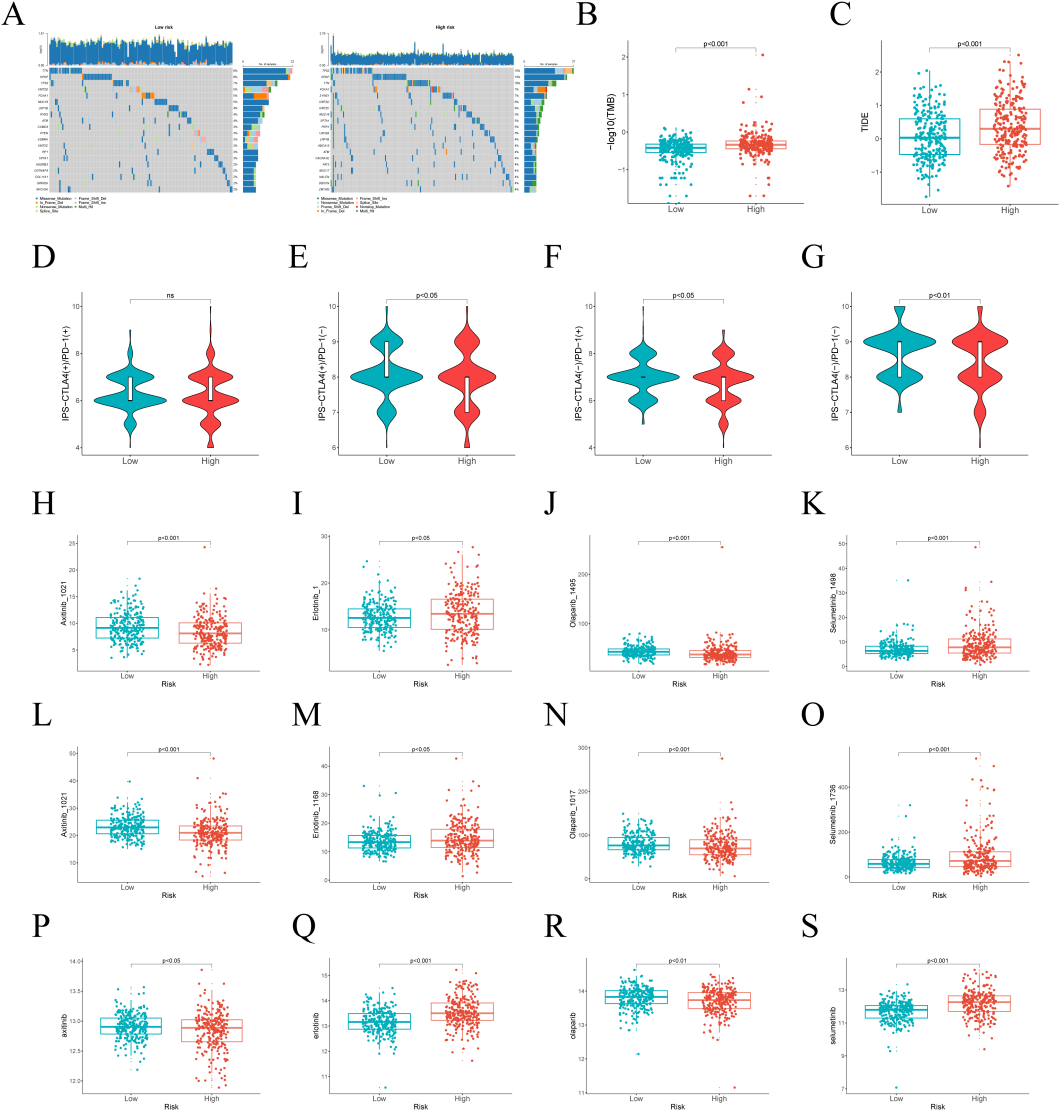
Differences in TMB and treatment response between high-risk and low-risk groups. **(A)** The top 20 genes with the highest mutation frequencies in risk groups. **(B)** Differences in TMB between different risk groups. **(C)** TIDE scores across various risk groups. **(D–G)** IPS in the high-risk and low-risk groups. **(H–S)** The predicted IC50 of Axitinib, Erlotinib, Olaparib, and Selumetinib in the GDSC 1.0, GDSC 2.0, and CTRP databases in two subgroups.

TMB analysis reveals distinct mutation profiles between risk groups, informing potential immunotherapy responses. Among the top 20 mutated genes, TP53 and SPOP predominated in high-risk patients, contrasting with TTN’s prevalence in low-risk cases (**Figure 6A**). Furthermore, TMB showed significant differences between the high-risk and low-risk groups (*p < 0.05*), suggesting that TMB may serve as a potential predictor of immunotherapy response (**Figure 6B**). Additionally, we performed TIDE scoring for both the high-risk and low-risk groups. The high-risk group exhibited higher TIDE scores, indicating a lower likelihood of benefiting from immunotherapy (**Figure 6C**). Moreover, IPS scoring revealed poor therapeutic responses to three immunotherapies in high-risk patients (**Figures 6D-G**). As chemotherapy remains the standard treatment for PCa, we utilized the GDSC 1.0, GDSC 2.0, and CTRP databases to analyze drug sensitivity and determine whether risk scores could reliably predict chemotherapy response. We assessed the sensitivity of Axitinib, Erlotinib, Olaparib, and Selumetinib across different risk groups (**Figures 6H**-**S**). The analysis indicated that Axitinib and Olaparib showed higher sensitivity in high-risk patients, while Erlotinib and Selumetinib showed higher sensitivity in low-risk group.

### Molecular docking and experiments to validate the function of DEGS1

**Figure 7:**
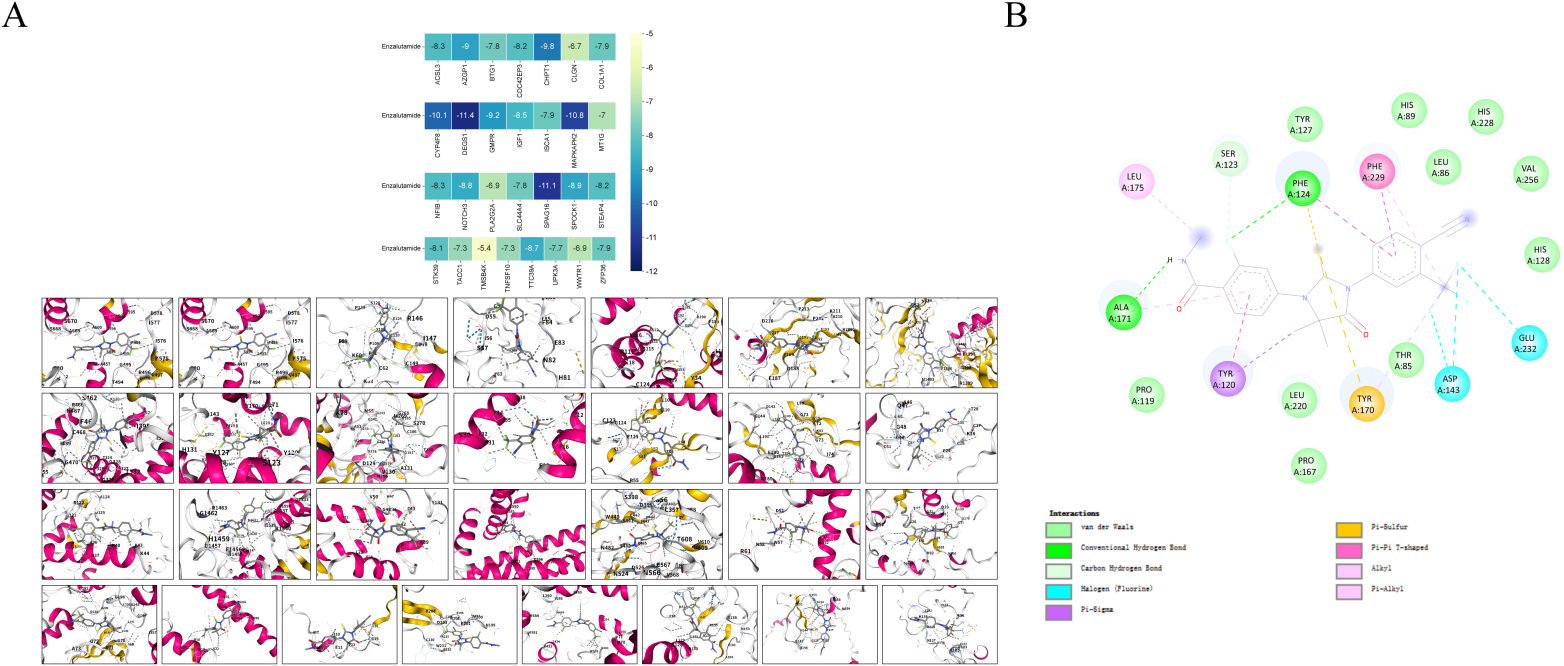
Molecular docking models and binding energy predictions of enzalutamide with 29 risk targets.

Based on 29 risk genes, molecular docking analysis was performed to undercover the potential binding interactions of enzalutamide with these proteins. The results demonstrated that enzalutamide could stably bind to the majority of the proteins. Notably, the most stable binding interactions were observed between enzalutamide and DEGS1 SPAG16, MAPKAPK2, CYP4F8, CHPT1, suggesting that these proteins may serve as potential therapeutic targets for enzalutamide (**Figure 7A**). Enzalutamide demonstrates strong and stable binding affinity to the DEGS1 protein, with a calculated binding energy of –11.4 kcal/mol. Specifically, its nitrogen and fluorine atoms form hydrogen bonds with Ala171 and Phe124, respectively, while the compound also engages in hydrophobic interactions with Tyr120, Tyr170, Leu175, and Phe229 (**Figure 7B**). Moreover, the trifluoromethyl group of enzalutamide forms halogen bonds with Asp143 and Glu232. Additionally, π–sulfur interactions involving Phe124 and Tyr170 further enhance the stability of the protein-ligand complex.

**Figure 8:**
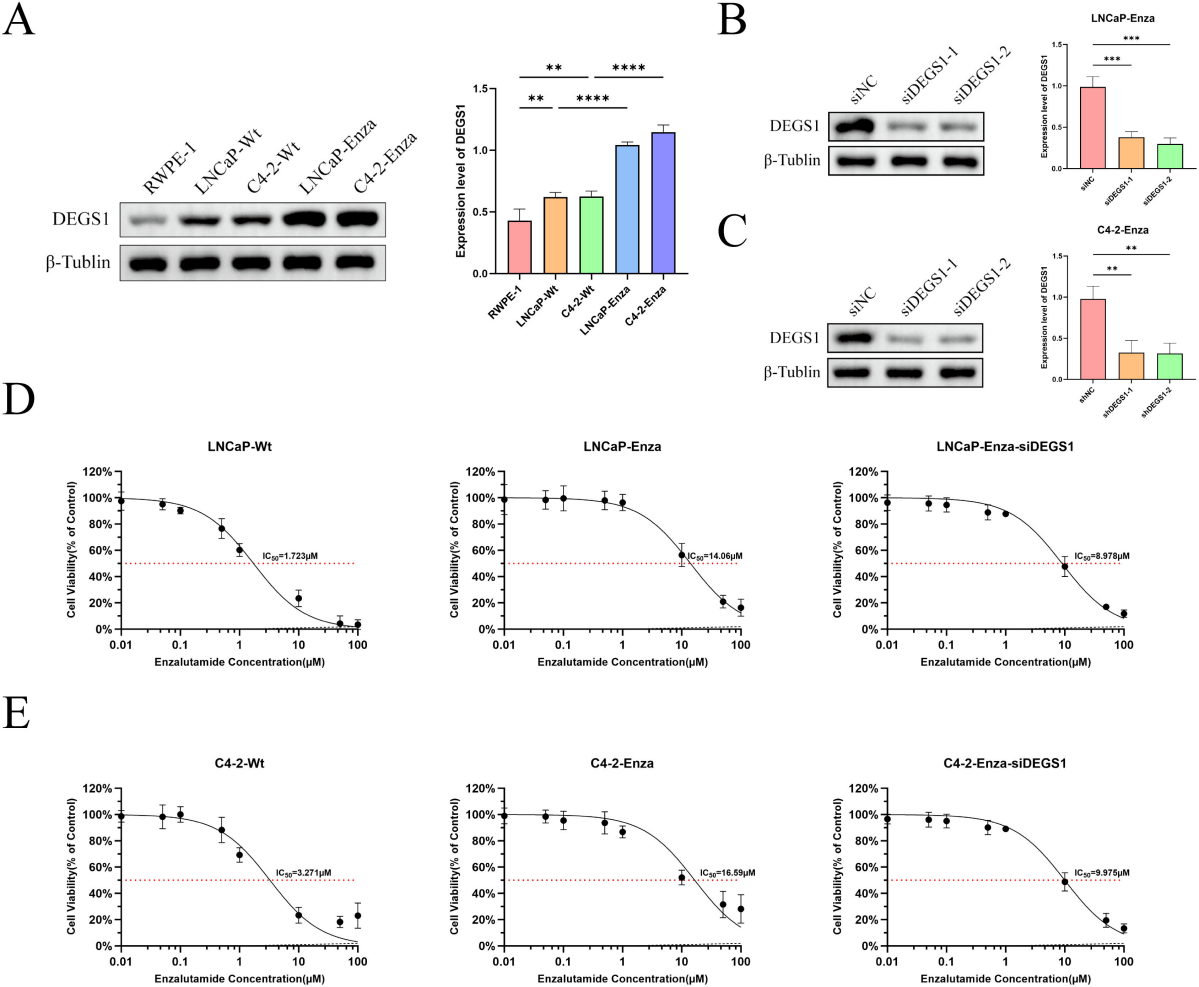
Expression levels of DEGS1 in various cell lines and its effect on the half-maximal inhibitory concentration (IC50) of enzalutamide. **(A)** DEGS1 expression levels in normal prostate epithelial cells (RWPE-1), prostate cancer cells (LNCaP, C4-2), and enzalutamide-resistant prostate cancer cells (LNCaP-Enza, C4-2-Enza). **(B, C)** Validation of DEGS1 knockdown efficiency following siRNA transfection. **(D, E)** The IC50 of prostate cancer cells and enzalutamide-resistant prostate cancer cells, the latter with or without siRNA transfection *p < 0.05, **p < 0.01, ***p < 0.001, ****p < 0.0001.

**Figure 9:**
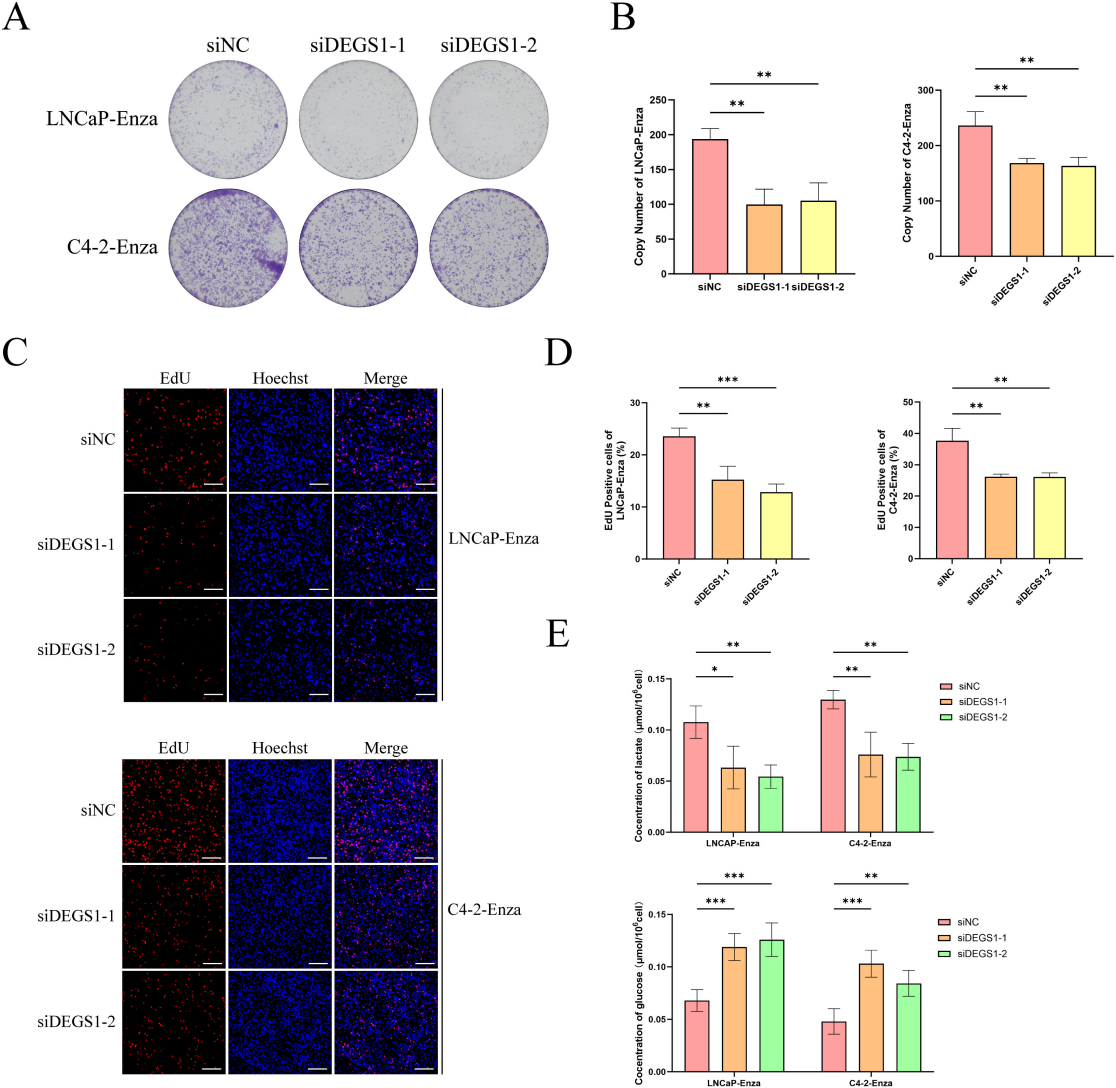
Impacts of DEGS1 on biological behaviors in prostate cancer cells. **(A, B)** Colony formation assays evaluating the proliferative capacity of cells. **(C, D)** EdU incorporation assays (red) assessing cell proliferation; nuclei were counterstained with Hoechst 33342 (blue). **(E)** Quantification of glucose consumption and lactate production among different treatment groups.

Multivariate Cox regression analysis combined with molecular docking studies identified DEGS1 as a gene potentially involved in the progression and drug resistance of prostate cancer (**Supplementary Figure 4**). To validate this finding, we assessed DEGS1 expression levels in prostate cancer cell lines. Western blot analysis revealed a significant upregulation of DEGS1 in enzalutamide-resistant cells (**Figure 8A**). To further elucidate the biological function of DEGS1, we silenced its expression using siRNA in LNCaP-Enza and C4-2-Enza cell lines (**Figures 8B, C**). We next assessed the enzalutamide resistance of LNCaP-Enza and C4-2-Enza cells after transfection with siRNA. Knockdown of DEGS1 significantly decreased the IC50 values of enzalutamide in both cell lines (**Figures 8D, E**), underscoring the role of DEGS1 in the acquisition of enzalutamide resistance in prostate cancer. Colony formation and EdU proliferation assays demonstrated that DEGS1 knockdown significantly suppressed the proliferative capacity of both cell lines (**Figures 9A-D**). Furthermore, analysis of intracellular glucose consumption and lactate production indicated that DEGS1 enhances glycolytic activity in prostate cancer cells and may mediate lactylation regulation (**Figure 9E**). Treatment of resistant cells with the LDH inhibitor Oxamate altered the expression of AR and its downstream targets, suggesting that lactylation may regulate AR activity and its associated signaling pathways (**Supplementary Figure 5**).

## Discussion

Various molecular features have been developed to predict tumor prognosis and treatment response, offering significant insights into tumor progression, cancer recurrence, and therapeutic outcomes (34, 35). Emerging evidence has progressively uncovered lactylation’s involvement in various pathological conditions, including cardiovascular disorders and inflammatory processes (36–38). Within oncological contexts, this epigenetic modification exerts dual regulatory effects, impacting both tumor cell metabolism and immune modulation through modulating the tumor microenvironment (39, 40).

This study integrated transcriptomic and single-cell sequencing to develop a prognostic model for PCa, incorporating lactylation-related genes, drug resistance-associated genes, and genes involved in the AR signaling pathway. Additionally, lactylation and drug resistance-related features were identified as potential predictors of progression-free interval PFI in PCa. The model’s stability was evaluated using multiple methodologies, and the correlations between risk genes, PFI, and clinical characteristics were thoroughly analyzed. Moreover, we compared genomic variations and drug sensitivity differences between the high-risk and low-risk groups, offering a comprehensive understanding of the clinical implications of the model. Finally, molecular docking analysis elucidated the binding patterns between enzalutamide and risk genes.

We employed univariate Cox regression and machine learning methods to identify 29 molecular features potentially associated with the progression of PCa, which may serve as protective or risk factors in its progression. Subsequently, based on multivariate Cox regression analysis, CDC42EP3, DEGS1, TACC1, and WWTR1 were identified as independent prognostic factors. Previous studies have suggested that these molecules may promote tumor progression and metastasis by influencing metabolic pathways or modulating the immune microenvironment (41–44).

ROC curves demonstrated that our prognostic model exhibited high accuracy, effectively distinguishing between low-risk and high-risk patients. KM curves indicated that the PFI was significantly longer in the low-risk group. The nomogram based on risk scores provided an intuitive visual tool for predicting PFI, while the calibration curve further confirmed the model’s robust performance in forecasting PFI.

Functional enrichment revealed potential regulatory mechanisms of prognostic features in PCa. GO and KEGG analyses indicated that differentially expressed genes between the high-risk and low-risk groups were significantly enriched in pathways related to hormone synthesis and neuroregulation. The nervous system regulates cellular functions by mediating neurotransmitter binding to receptors on target cells. During PCa progression, it may undergo neuroendocrine transformation, leading to the development of highly invasive and castration-resistant neuroendocrine prostate cancer. Existing studies suggest that metabolic reprogramming in PCa increases lactylation levels, which, in turn, upregulate neuroendocrine-associated genes in tumors (13, 45, 46). Moreover, resistance mechanisms in PCa partly arise from increased intratumoral synthesis of androgens and steroid hormones. This includes enhanced overall androgen synthesis and an increased conversion of dehydroepiandrosterone sulfate (DHEA-S) to dehydroepiandrosterone (DHEA) in the adrenal gland. These alterations reduce the effectiveness of androgen receptor signaling inhibitors (ARSI) and contribute to drug resistance. Another factor in resistance is the conformational changes induced by point mutations in the AR, which activate androgen-independent AR downstream signaling pathways (47, 48). GSEA also suggested that lactylation may promote increased hormone synthesis while inhibiting androgen response. This indicates that lactylation could influence PCa progression by modulating androgen levels or AR activity, and our experimental data provide validation of this mechanism (**Supplementary Figure 5**).

We evaluated the drug treatment response in both two groups of patients. The TIDE score revealed that high-risk patients are more susceptible to immune escape, whereas the IPS score indicated a lower response rate to immunotherapy in this group. Previous studies have demonstrated that lactate produced by tumor cells can impair the phagocytic activity of activated macrophages, induce apoptosis in natural killer (NK) cells and natural killer T (NKT) cells, and inhibit cytokine release, thus contributing to extensive immunosuppressive effects within the tumor microenvironment (TME) (49–51). Histone lactylation, including H3K18 lactylation, inhibits CD8+ T cell function within the tumor microenvironment, facilitating immune evasion (8, 51). Furthermore, Enrichment analysis identified significantly elevated steroid hormone levels in the high-risk group, potentially contributing to tumor microenvironment modulation. This finding aligns with established evidence that androgen pathway inhibition enhances tumor sensitivity to both immunotherapy and targeted treatment regimens (52). Analysis of chemotherapy drug sensitivity showed that the therapeutic efficacy of Erlotinib and Selumetinib is likely to be better efficacy in the high-risk group. These findings highlight the importance of the predictive model in customizing treatment strategies for patients, enabling the identification of individuals most likely to benefit from immunotherapy and targeted therapies, thereby advancing the application of precision medicine.

Finally, we identified the potential therapeutic targets of enzalutamide through molecular docking analysis. The results demonstrated that enzalutamide could stably bind to most of the risk-associated target proteins. Notably, the interactions with DEGS1 SPAG16, MAPKAPK2, CYP4F8 and CHPT1 exhibited the lowest binding energies, indicating the most stable binding interactions. These findings suggest that these proteins could serve as promising therapeutic targets or key molecules for reversing tumor resistance. Integrating previous results of multivariate Cox analysis, DEGS1 may plays a key role in progression and drug resistance in prostate cancer. To elucidate the biological function of DEGS1 in prostate cancer progression, we conducted a series of experiments. Western blot analysis demonstrated that DEGS1 expression was markedly upregulated in enzalutamide-resistant prostate cancer cells. EdU and colony formation assays confirmed that DEGS1 facilitates the proliferation of these resistant cells. Additionally, assessments of glucose consumption and lactate production revealed that DEGS1 enhances glycolytic activity, implying a potential role in promoting lactylation.

Despite the promising outcomes of this study, several limitations must be addressed. First, the mechanistic underpinnings of the identified risk genes necessitate further experimental validation. Our findings confirm the pivotal role of DEGS1 in glucose metabolism regulation, as its knockout markedly diminishes intracellular glucose consumption and lactate production. Nevertheless, its influence on lactylation modification remains unresolved and warrants further investigation. This prognostic model exhibits robust predictive accuracy, its clinical translation necessitates additional validation. The retrospective design and exclusive dependence on public genomic databases, without prospective multicenter cohort validation, may limit the generalizability of our findings. Furthermore, the modest sample size (n=4) in our single-cell RNA sequencing analysis warrants cautious interpretation. Although our integrated computational and *in vitro* approaches including molecular docking and cell-based assays have substantiated DEGS1’s functional involvement in enzalutamide resistance, com prehensive *in vivo* validation remains imperative to establish its therapeutic relevance. Additionally, the molecular docking results are theoretical and may overlook other functionally significant molecules that exhibit slightly higher but still biologically relevant binding affinities. For instance, SPAG16, MAPKAPK2, and CYP4F8 all demonstrated substantial binding energies that could still mediate important pharmacological interactions. Future research should incorporate integrated multi-omics approaches, particularly spatial transcriptomics, to elucidate the cellular heterogeneity of lactylation modifications at single-cell resolution. The application of CRISPR-based screening coupled with organoid culture systems would enable functional validation of critical genes involved in lactylation-mediated drug resistance mechanisms. Furthermore, comprehensive cross-cancer multi-omics analyses are essential to systematically delineate the relationship between lactylation regulatory networks and therapeutic outcomes. Such multidimensional investigations would yield more conclusive evidence to support the development of precision medicine strategies.

## Conclusion

This study successfully developed a tumor prognostic model with high predictive accuracy by integrating lactylation-related gene sets with machine learning techniques, and its clinical relevance was validated through comprehensive analysis. The results demonstrated that lactylation-associated risk features not only impact the survival prognosis of cancer patients but also have the potential to influence treatment outcomes by modulating tumor metabolism and the immune microenvironment. Significant differences were observed between high- and low-risk groups regarding immune escape potential, immunotherapy response, and chemotherapy drug sensitivity, offering valuable insights for personalized therapeutic strategies. Additionally, molecular docking analysis revealed stable binding interactions between enzalutamide and risk gene proteins, suggesting that these genes may represent viable therapeutic targets. Collectively, these findings provide novel perspectives on the molecular mechanisms of lactylation modifications in tumors and lay a theoretical foundation for tumor survival prediction and personalized treatment approaches.

## Data Availability

The TCGA cohort data are publicly available and can be obtained from the UCSC Xena platform (https://xenabrowser.net). SNV data were sourced from The Cancer Genome Atlas (https://portal.gdc.cancer.gov). The single-cell dataset GSE206962 was downloaded from the GEO database (https://www.ncbi.nlm.nih.gov/geo). Four independent datasets (GSE54460, DKFZ, Stockholm, and Taylor) are freely available for download via the PCaDB database (http://bioinfo.jialab-ucr.org/PCaDB). The data associated with TIDE and IPS are derived from the TIDE Database (http://tide.dfci.harvard.edu) and the TCIA Database (https://tcia.at/home), respectively. Additional data related to this study are available upon reasonable request from the corresponding authors.

## References

[1] Bray F Laversanne M Sung H Ferlay J Siegel RL Soerjomataram I. Global cancer statistics 2022: GLOBOCAN estimates of incidence and mortality worldwide for 36 cancers in 185 countries. CA Cancer J Clin. (2024) 74:229–63. doi: 10.3322/caac.21834, PMID: 38572751

[2] Dai C Heemers H Sharifi N. Androgen Signaling in Prostate Cancer. Cold Spring Harb Perspect Med. (2017) 7. doi: 10.1101/cshperspect.a030452, PMID: 28389515 PMC5580512

[3] Yap TA Smith AD Ferraldeschi R Al-Lazikani B Workman P de Bono JS. Drug discovery in advanced prostate cancer: translating biology into therapy. Nat Rev Drug Discov. (2016) 15:699–718. doi: 10.1038/nrd.2016.120, PMID: 27444228

[4] Wadosky KM Koochekpour S. Molecular mechanisms underlying resistance to androgen deprivation therapy in prostate cancer. Oncotarget. (2016) 7:64447–70. doi: 10.18632/oncotarget.10901, PMID: 27487144 PMC5325456

[5] Huber F Montani M Sulser T Jaggi R Wild P Moch H. Comprehensive validation of published immunohistochemical prognostic biomarkers of prostate cancer -what has gone wrong? A blueprint for the way forward in biomarker studies. Br J Cancer. (2015) 112:140–8. doi: 10.1038/bjc.2014.588, PMID: 25422912 PMC4453620

[6] Harlan SR Cooperberg MR Elkin E Lubeck DP Meng M Mehta SS. Time trends and characteristics of men choosing watchful waiting for initial treatment of localized prostate cancer: results from CaPSURE. J Urol. (2003) 170:1804–7. doi: 10.1097/01.ju.0000091641.34674.11, PMID: 14532780

[7] Luo Y Zhang N Ye J Wang Z Zhou X Liu J. Unveiling lactylation modification: A new hope for cancer treatment. BioMed Pharmacother. (2025) 184:117934. doi: 10.1016/j.biopha.2025.117934, PMID: 39986235

[8] Ma Z Yang J Jia W Li L Li Y Hu J. Histone lactylation-driven B7-H3 expression promotes tumor immune evasion. Theranostics. (2025) 15:2338–59. doi: 10.7150/thno.105947, PMID: 39990209 PMC11840737

[9] Wu Z Peng Y Chen W Xia F Song T Ke Q. Lactylation-driven transcriptional activation of FBXO33 promotes gallbladder cancer metastasis by regulating p53 polyubiquitination. Cell Death Dis. (2025) 16:144. doi: 10.1038/s41419-025-07372-y, PMID: 40021626 PMC11871038

[10] Li G Wang D Zhai Y Pan C Zhang J Wang C. Glycometabolic reprogramming-induced XRCC1 lactylation confers therapeutic resistance in ALDH1A3-overexpressing glioblastoma. Cell Metab. (2024) 36:1696–710.e10. doi: 10.1016/j.cmet.2024.07.011, PMID: 39111285

[11] Chen J Huang Z Chen Y Tian H Chai P Shen Y. Lactate and lactylation in cancer. Signal Transduct Target Ther. (2025) 10:38. doi: 10.1038/s41392-024-02082-x, PMID: 39934144 PMC11814237

[12] Jing F Zhang J Zhang H Li T. Unlocking the multifaceted molecular functions and diverse disease implications of lactylation. Biol Rev Camb Philos Soc. (2025) 100:172–89. doi: 10.1111/brv.13135, PMID: 39279350

[13] Wang D Du G Chen X Wang J Liu K Zhao H. Zeb1-controlled metabolic plasticity enables remodeling of chromatin accessibility in the development of neuroendocrine prostate cancer. Cell Death Differ. (2024) 31:779–91. doi: 10.1038/s41418-024-01295-5, PMID: 38654072 PMC11164927

[14] Chen H Li Y Li H Chen X Fu H Mao D. NBS1 lactylation is required for efficient DNA repair and chemotherapy resistance. Nature. (2024) 631:663–9. doi: 10.1038/s41586-024-07620-9, PMID: 38961290 PMC11254748

[15] Niu K Chen Z Li M Ma G Deng Y Zhang J. NSUN2 lactylation drives cancer cell resistance to ferroptosis through enhancing GCLC-dependent glutathione synthesis. Redox Biol. (2025) 79:103479. doi: 10.1016/j.redox.2024.103479, PMID: 39742570 PMC11750563

[16] Yang R He J Kang D Chen Y Huang J Li J. Bioinformatics analysis reveals novel tumor antigens and immune subtypes of skin cutaneous melanoma contributing to mRNA vaccine development. Front Immunol. (2025) 16:1520505. doi: 10.3389/fimmu.2025.1520505, PMID: 40066453 PMC11891200

[17] Peng Y Yang J Ao J Li Y Shen J He X. Single-cell profiling reveals the intratumor heterogeneity and immunosuppressive microenvironment in cervical adenocarcinoma. Elife. (2025) 13. doi: 10.7554/eLife.97335, PMID: 40066698 PMC11896611

[18] Hashemi Karoii D Shakeri Abroudi A Forghani N Bavandi S Djamali M Baghaei H. Analysis of microarray and single-cell RNA-seq identifies gene co-expression, cell-cell communication, and tumor environment associated with metabolite interconversion enzyme in prostate cancer. Discov Oncol. (2025) 16:177. doi: 10.1007/s12672-025-01926-4, PMID: 39946051 PMC11825437

[19] Liu J Lichtenberg T Hoadley KA Poisson LM Lazar AJ Cherniack AD. An Integrated TCGA Pan-Cancer Clinical Data Resource to Drive High-Quality Survival Outcome Analytics. Cell. (2018) 173:400–16.e11. doi: 10.1016/j.cell.2018.02.052, PMID: 29625055 PMC6066282

[20] Butler A Hoffman P Smibert P Papalexi E Satija R. Integrating single-cell transcriptomic data across different conditions, technologies, and species. Nat Biotechnol. (2018) 36:411–20. doi: 10.1038/nbt.4096, PMID: 29608179 PMC6700744

[21] Chen Z Li Y Yuan Y Lai K Ye K Lin Y. Single-cell sequencing reveals homogeneity and heterogeneity of the cytopathological mechanisms in different etiology-induced AKI. Cell Death Dis. (2023) 14:318. doi: 10.1038/s41419-023-05830-z, PMID: 37169762 PMC10175265

[22] Liu C Liu J Yang Y. Bulk and Single-Cell Transcriptomic Reveals Shared Key Genes and Patterns of Immune Dysregulation in Both Intestinal Inflammatory Disease and Sepsis. J Cell Mol Med. (2025) 29:e70415. doi: 10.1111/jcmm.70415, PMID: 39993996 PMC11850196

[23] Yang Y Chen X Pan J Ning H Zhang Y Bo Y. Pan-cancer single-cell dissection reveals phenotypically distinct B cell subtypes. Cell. (2024) 187:4790–811.e22. doi: 10.1016/j.cell.2024.06.038, PMID: 39047727

[24] Yang Y Liu Z Wei Y He S Gu A Li Z. Single-cell multi-omics analysis reveals candidate therapeutic drugs and key transcription factor specifically for the mesenchymal subtype of glioblastoma. Cell Biosci. (2024) 14:151. doi: 10.1186/s13578-024-01332-3, PMID: 39707474 PMC11662595

[25] Engebretsen S Bohlin J. Statistical predictions with glmnet. Clin Epigenetics. (2019) 11:123. doi: 10.1186/s13148-019-0730-1, PMID: 31443682 PMC6708235

[26] Wu J Zhang H Li L Hu M Chen L Xu B. A nomogram for predicting overall survival in patients with low-grade endometrial stromal sarcoma: A population-based analysis. Cancer Commun (Lond). (2020) 40:301–12. doi: 10.1002/cac2.12067, PMID: 32558385 PMC7365459

[27] Wu T Hu E Xu S Chen M Guo P Dai Z. clusterProfiler 4.0: A universal enrichment tool for interpreting omics data. Innovation (Camb). (2021) 2:100141. doi: 10.1016/j.xinn.2021.100141, PMID: 34557778 PMC8454663

[28] Mayakonda A Lin DC Assenov Y Plass C Koeffler HP. Maftools: efficient and comprehensive analysis of somatic variants in cancer. Genome Res. (2018) 28:1747–56. doi: 10.1101/gr.239244.118, PMID: 30341162 PMC6211645

[29] Charoentong P Finotello F Angelova M Mayer C Efremova M Rieder D. Pan-cancer Immunogenomic Analyses Reveal Genotype-Immunophenotype Relationships and Predictors of Response to Checkpoint Blockade. Cell Rep. (2017) 18:248–62. doi: 10.1016/j.celrep.2016.12.019, PMID: 28052254

[30] Yang W Soares J Greninger P Edelman EJ Lightfoot H Forbes S. Genomics of Drug Sensitivity in Cancer (GDSC): a resource for therapeutic biomarker discovery in cancer cells. Nucleic Acids Res. (2013) 41:D955–61. doi: 10.1093/nar/gks1111, PMID: 23180760 PMC3531057

[31] Basu A Bodycombe NE Cheah JH Price EV Liu K Schaefer GI. An interactive resource to identify cancer genetic and lineage dependencies targeted by small molecules. Cell. (2013) 154:1151–61. doi: 10.1016/j.cell.2013.08.003, PMID: 23993102 PMC3954635

[32] Zhang Y Li Z Wei J Kong L Song M Zhang Y. Network pharmacology and molecular docking reveal the mechanism of Angelica dahurica against Osteosarcoma. Med (Baltimore). (2022) 101:e31055. doi: 10.1097/md.0000000000031055, PMID: 36343039 PMC9646661

[33] Tang D Chen M Huang X Zhang G Zeng L Zhang G. SRplot: A free online platform for data visualization and graphing. PloS One. (2023) 18:e0294236. doi: 10.1371/journal.pone.0294236, PMID: 37943830 PMC10635526

[34] Guo S Lv G Zhu H Guo Y Yin K Yu H. Disulfidptosis related immune genes drive prognostic model development and tumor microenvironment characterization in bladder urothelial carcinoma. Sci Rep. (2025) 15:8130. doi: 10.1038/s41598-025-92297-x, PMID: 40057601 PMC11890603

[35] Fu C Sun L Feng C Zhou T Bi Y. A prognostic model of lung adenocarcinoma constructed based on circadian rhythm genes and its potential clinical significance. Front Oncol. (2025) 15:1464578. doi: 10.3389/fonc.2025.1464578, PMID: 40040723 PMC11876053

[36] Chen Z Zhong M Lin Y Zhang W Zhu Y Chen L. METTL7B-induced histone lactylation prevents heart failure by ameliorating cardiac remodelling. J Mol Cell Cardiol. (2025). doi: 10.1016/j.yjmcc.2025.03.006, PMID: 40068772

[37] Wang S Zheng H Zhao J Xie J. Role of lysine lactylation in neoplastic and inflammatory pulmonary diseases (Review). Int J Mol Med. (2025) 55. doi: 10.3892/ijmm.2025.5512, PMID: 40052587 PMC11913435

[38] Peng TY Lu JM Zheng XL Zeng C He YH. The role of lactate metabolism and lactylation in pulmonary arterial hypertension. Respir Res. (2025) 26:99. doi: 10.1186/s12931-025-03163-3, PMID: 40075458 PMC11905457

[39] Gong H Nie D Li Z. The crosstalk between broad epigenetic modification and T cell metabolism within tumor microenvironment. Int Immunopharmacol. (2025) 152:114410. doi: 10.1016/j.intimp.2025.114410, PMID: 40068521

[40] Lv M Huang Y Chen Y Ding K. Lactylation modification in cancer: mechanisms, functions, and therapeutic strategies. Exp Hematol Oncol. (2025) 14:32. doi: 10.1186/s40164-025-00622-x, PMID: 40057816 PMC11889934

[41] Yan Y Liang Q Xu Z Yi Q. Integrative bioinformatics and experimental analysis revealed down-regulated CDC42EP3 as a novel prognostic target for ovarian cancer and its roles in immune infiltration. PeerJ. (2021) 9:e12171. doi: 10.7717/peerj.12171, PMID: 34616622 PMC8449529

[42] Zhang X Zhao H Li Y Xia D Yang L Ma Y. The role of YAP/TAZ activity in cancer metabolic reprogramming. Mol Cancer. (2018) 17:134. doi: 10.1186/s12943-018-0882-1, PMID: 30176928 PMC6122186

[43] Mu H Hu J Lin Z Wei L Li Q Wang X. Integration of network pharmacology, metabolomics and lipidomics for clarifying the role of sphingolipid metabolism in the treatment of liver cancer by regorafenib. Life Sci. (2024) 358:123165. doi: 10.1016/j.lfs.2024.123165, PMID: 39447728

[44] Vendrell JA Magnino F Danis E Duchesne MJ Pinloche S Pons M. Estrogen regulation in human breast cancer cells of new downstream gene targets involved in estrogen metabolism, cell proliferation and cell transformation. J Mol Endocrinol. (2004) 32:397–414. doi: 10.1677/jme.0.0320397, PMID: 15072547

[45] Romero R Chu T González Robles TJ Smith P Xie Y Kaur H. The neuroendocrine transition in prostate cancer is dynamic and dependent on ASCL1. Nat Cancer. (2024) 5:1641–59. doi: 10.1038/s43018-024-00838-6, PMID: 39394434 PMC11584404

[46] He Y Ji Z Gong Y Fan L Xu P Chen X. Numb/Parkin-directed mitochondrial fitness governs cancer cell fate via metabolic regulation of histone lactylation. Cell Rep. (2023) 42:112033. doi: 10.1016/j.celrep.2023.112033, PMID: 36724072

[47] Khorasanchi A Hong F Yang Y Singer EA Wang P Li M. Overcoming drug resistance in castrate-resistant prostate cancer: current mechanisms and emerging therapeutic approaches. Cancer Drug Resist. (2025) 8:9. doi: 10.20517/cdr.2024.173, PMID: 40051495 PMC11883235

[48] Li C Cheng D Li P. Androgen receptor dynamics in prostate cancer: from disease progression to treatment resistance. Front Oncol. (2025) 15:1542811. doi: 10.3389/fonc.2025.1542811, PMID: 40008000 PMC11850250

[49] Chaudagar K Hieromnimon HM Khurana R Labadie B Hirz T Mei S. Reversal of Lactate and PD-1-mediated Macrophage Immunosuppression Controls Growth of PTEN/p53-deficient Prostate Cancer. Clin Cancer Res. (2023) 29:1952–68. doi: 10.1158/1078-0432.Ccr-22-3350, PMID: 36862086 PMC10192075

[50] Zhang Y Peng Q Zheng J Yang Y Zhang X Ma A. The function and mechanism of lactate and lactylation in tumor metabolism and microenvironment. Genes Dis. (2023) 10:2029–37. doi: 10.1016/j.gendis.2022.10.006, PMID: 37492749 PMC10363641

[51] Zhang C Zhou L Zhang M Du Y Li C Ren H. H3K18 Lactylation Potentiates Immune Escape of Non-Small Cell Lung Cancer. Cancer Res. (2024) 84:3589–601. doi: 10.1158/0008-5472.Can-23-3513, PMID: 39137401

[52] Li F Xing X Jin Q Wang XM Dai P Han M. Sex differences orchestrated by androgens at single-cell resolution. Nature. (2024) 629:193–200. doi: 10.1038/s41586-024-07291-6, PMID: 38600383

