# Supplementary material for: Integrative single-cell and machine learning analysis predicts lactylation-driven therapy resistance in prostate cancer: a molecular docking and experiments-validated framework for treatment optimization
Source: Front Immunol. 2025 Sep 2;16:1647384. doi: 10.3389/fimmu.2025.1647384 (PMC12436506; doi:10.3389/fimmu.2025.1647384)
Supplement: Supplementary file 1 [file DataSheet1.docx]

**Supplementary Figures**

**Supplemental Figure1**

| 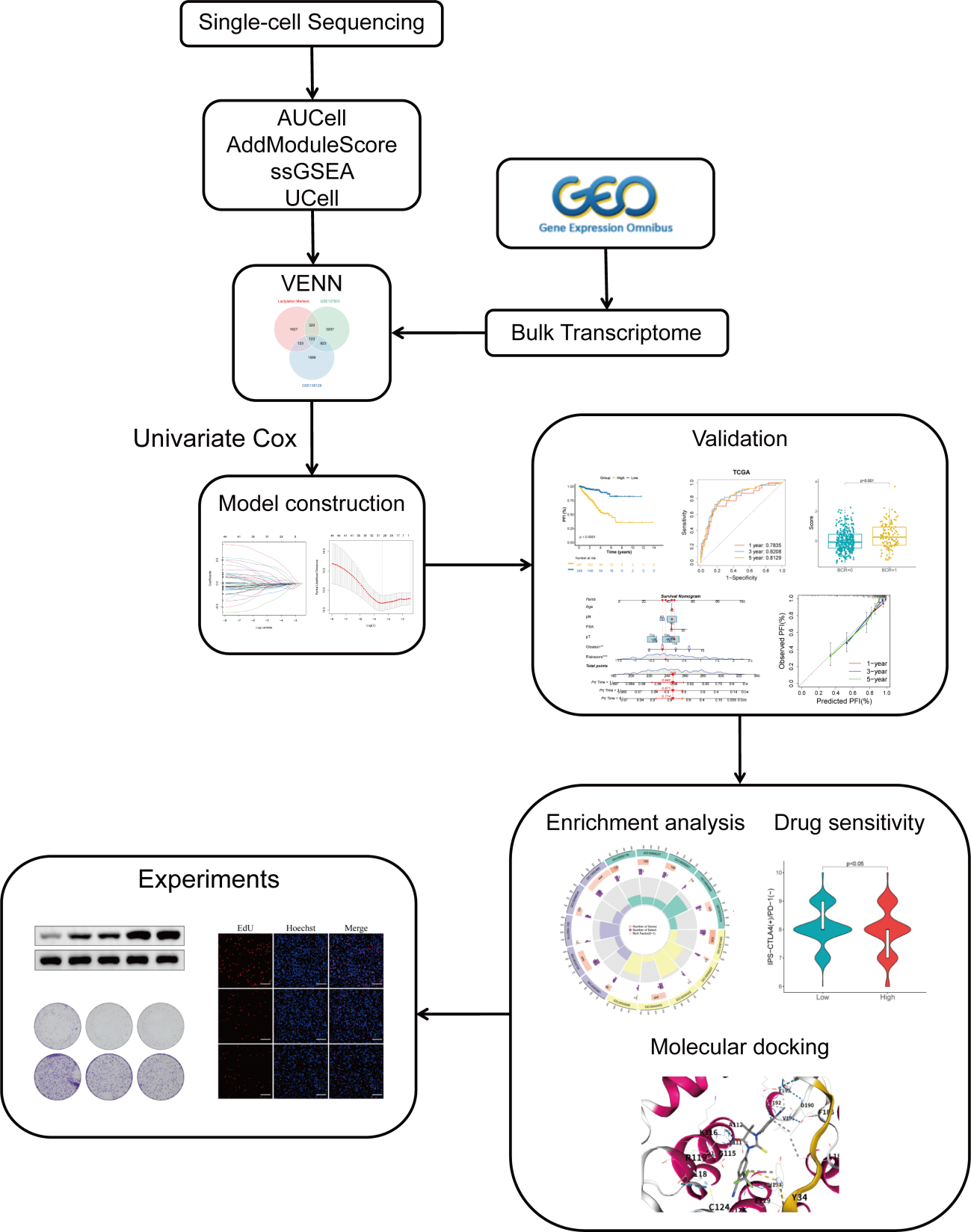 |
| --- |

**Supplementary Fig.1 The flowchart of this study.**

**Supplementary Figure2**

| **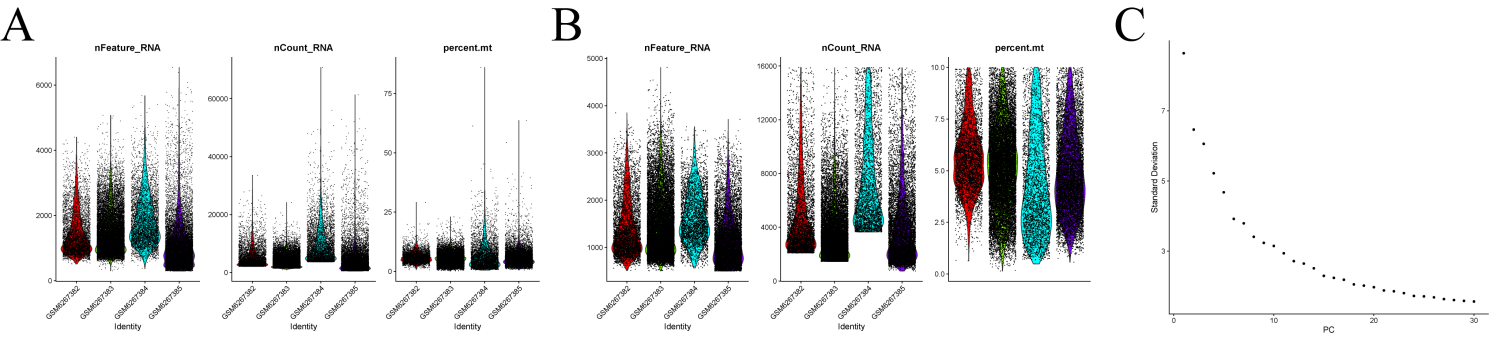** |
| --- |

**Supplementary Fig. 2 Quality control of single-cell RNA sequencing.**

A. Violin plot prior to quality control. B. Violin plot after quality control. C. Elbow plot after quality control.

**Supplementary Figure3**

| 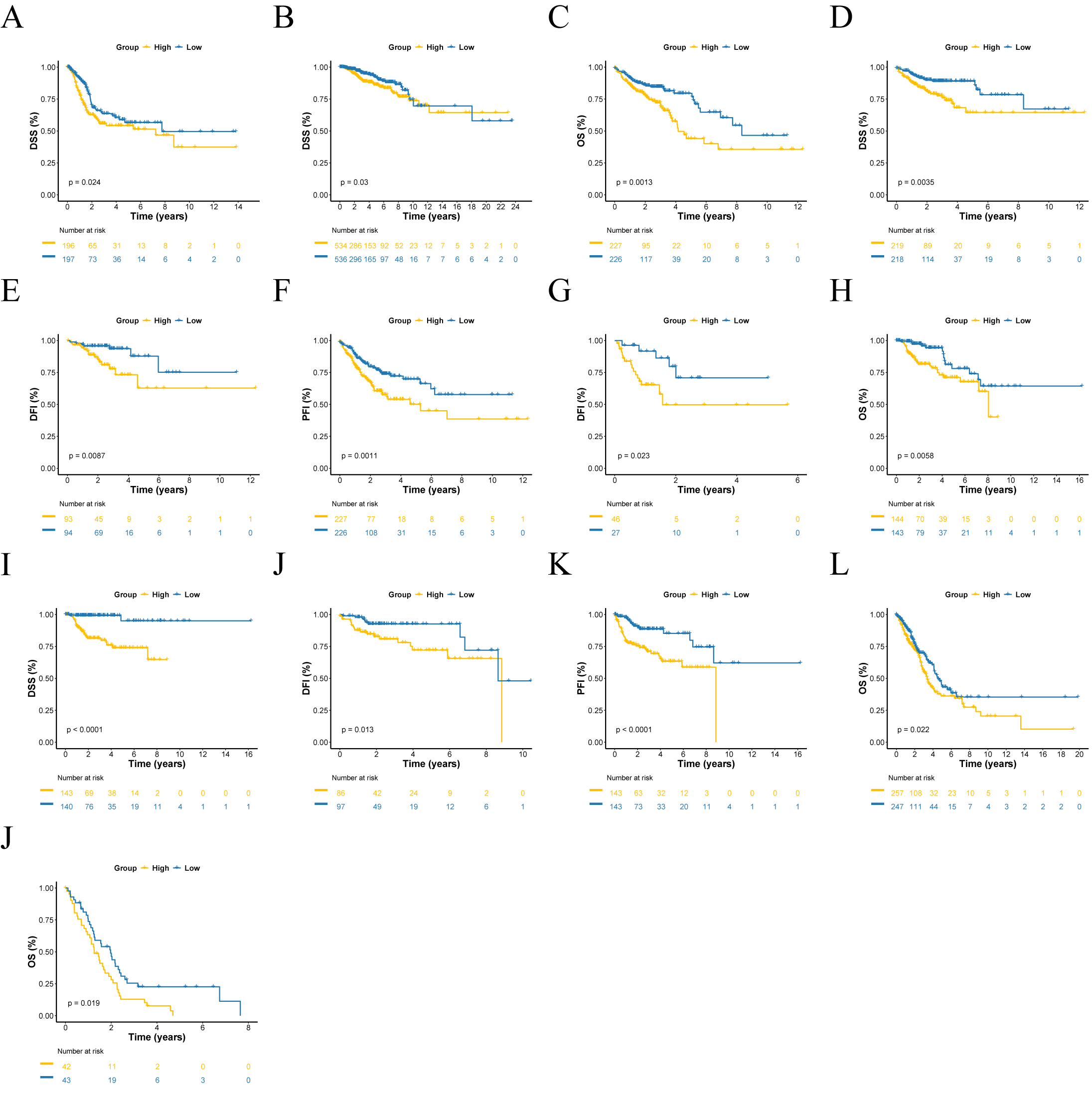 |
| --- |

**Supplementary Fig.3 Survival curves predicting prognosis for different risk groups in various tumors**A. KM curve validating the predictive performance of the risk score in BLCA. B. KM curve demonstrating the predictive performance of the risk score in BRCA. C-F. KM curves used to validate the predictive performance of the risk score in COAD. G. KM curve used to validate the predictive performance of the risk score in ESCA. H-K. KM curves validating the predictive performance of the risk score in KIRP. L. KM curve validates the predictive performance of the risk score in LUAD. J. KM curve demonstrates the predictive performance of the risk score in MESO.

**Supplementary Figure4**

| 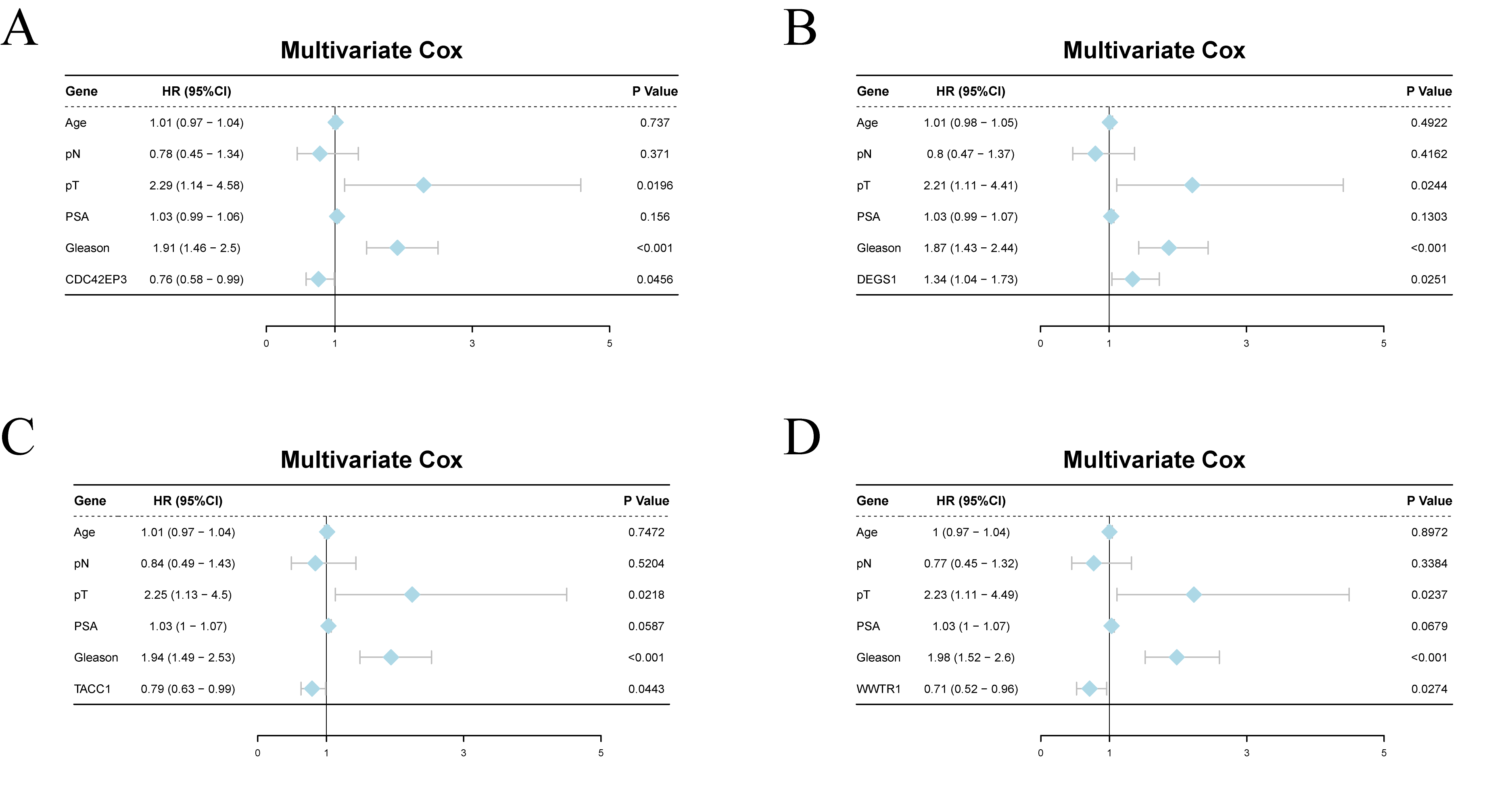 |
| --- |

**Supplementary Fig.4 Multivariate Cox regression analysis on 4 risk genes and clinical features on forest plots.**

**Supplementary Figure5**

| **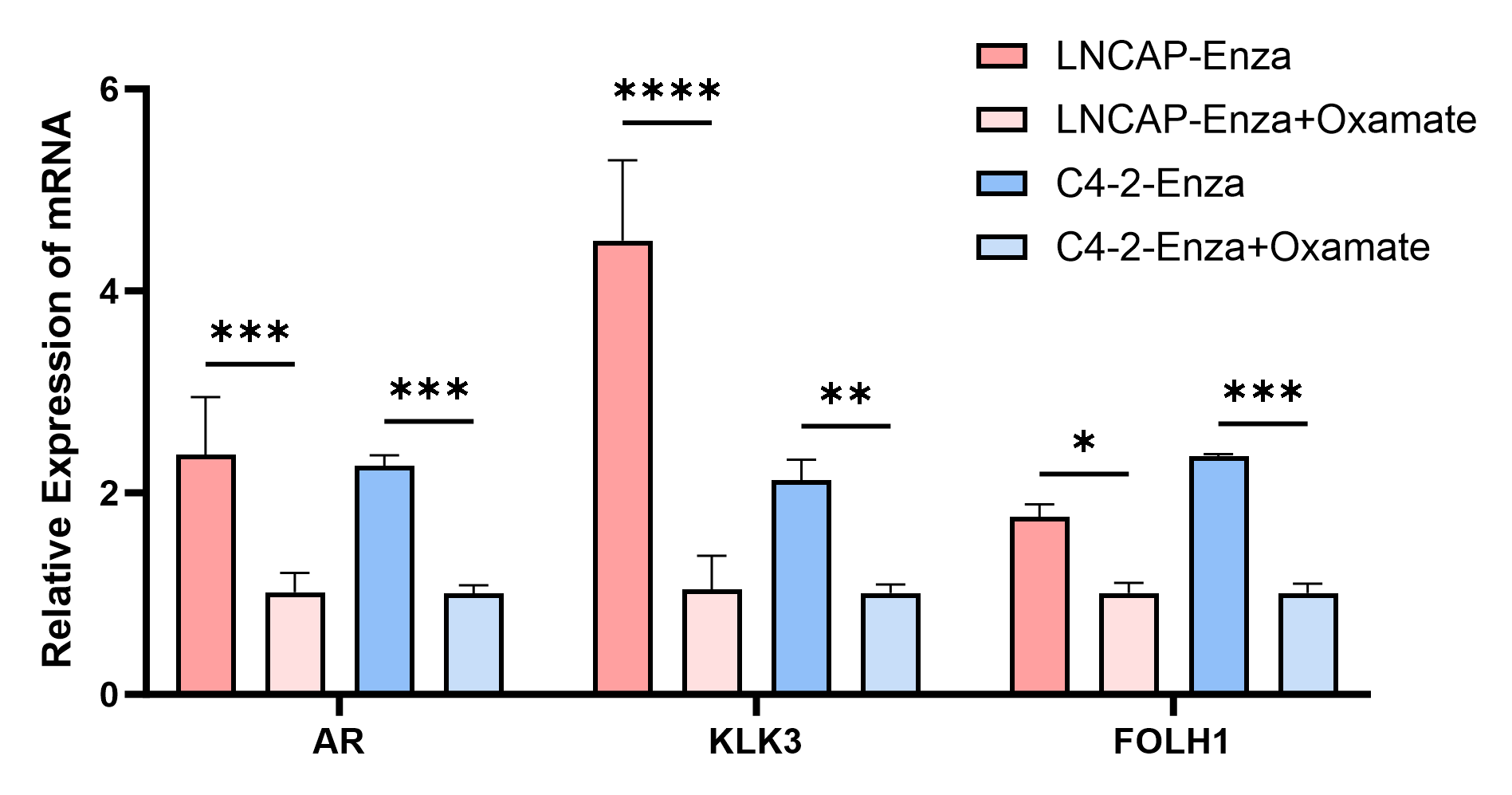** |
| --- |

**Supplementary Fig.5 RT-qPCR was performed to detect the expression of AR and its downstream target genes in enzalutamide-resistant cell lines with/without Oxamate treatment.**
